# Salt Stress Differentially Affects the Primary and Secondary Metabolism of Peppers (*Capsicum annuum* L.) According to the Genotype, Fruit Part, and Salinity Level

**DOI:** 10.3390/plants11070853

**Published:** 2022-03-23

**Authors:** Tilen Zamljen, Aljaz Medic, Metka Hudina, Robert Veberic, Ana Slatnar

**Affiliations:** Department of Agronomy, Biotechnical Faculty, University of Ljubljana, SI-1000 Ljubljana, Slovenia; aljaz.medic@bf.uni-lj.si (A.M.); metka.hudina@bf.uni-lj.si (M.H.); robert.veberic@bf.uni-lj.si (R.V.); ana.slatnar@bf.uni-lj.si (A.S.)

**Keywords:** abiotic stress, capsaicinoids, hydroponics, phenolics

## Abstract

A total of four *Capsicum annuum* L. genotypes (‘Caro F1’, ‘Berenyi F1’, ‘Somborka’ and ‘Novosadka’) were exposed to two intensities of salt stress. We observed a significant decrease in the sugar content in all salt stressed treatments, except for the sucrose content of the pericarp of the ‘Caro F1’ cultivar. Salt stress had a largely negative effect on the total and individual organic acid content, although the effect differed among cultivars. Using high performance liquid chromatography coupled with a mass spectrometer, most phenolics were identified in the pericarp (18), followed by the placenta (7) and seeds (8). Treatment with 40 mM NaCl caused the highest increase in individual phenols, followed by treatment with 20 mM NaCl. The cultivar ‘Berenyi F1’ was less affected by salt stress treatment than the other three cultivars in terms of content of individual and total phenols. Salt stress increased the content of capsaicinoids in all the cultivars. The pericarp of the cultivar ‘Novosadka’ showed 17.5 and 50 times higher total capsaicinoid content than the control in the 20 mM and 40 mM NaCl, respectively. With the results of several metabolite groups, we confirmed that the reaction and metabolic content to salt stress within the genus *Capsicum* is genotype-, fruit part-, and salinity level-dependent.

## 1. Introduction

Pungent and non-pungent peppers are an important crop worldwide, being grown for fresh consumption, spice, ornamentation, or medicinal purposes [1]. There are around 30 different species, with *Capsicum annuum* L. being the most widely spread and cultivated [2]. They are native to the tropical and subtropical Americas and thus susceptible to various abiotic stresses [3,4,5]. Chilies and peppers are usually grown in greenhouses whereby constant irrigation is required to ensure optimum water availability. The fertilizer requirement is high in chilies, so mineral fertilizer is usually used to meet the nutrient requirements [6]. Irrigation and fertilization can lead to increased soil salinity [7].

Soil salinization is an abiotic stress for plants and a major problem in agriculture [4], affecting more than 100 countries or 23% of the total cultivated area [7]. Salinity is independent of climatic conditions, which means that it can occur in any type of environment, the most in arid and semi-arid regions where there is insufficient rainfall to meet the water needs of crops, so irrigation is required. When irrigation is combined with poor drainage, soil salinity can increase rapidly [7,8].

Sodium chloride (NaCl) is particularly problematic for soil salinity, causing slower growth, leaf senescence, reduced plant branching, and lower yields. The sodium ion (Na^+^) in high concentrations causes great damage to the cytosol of leaf cells since it interferes with many metabolic processes such as photosynthesis [9]. Salt stress induces the formation of cytotoxically-activated oxygen, which causes oxidative damage to lipids, proteins, and nucleic acids [10]. In addition, salinity can cause the formation of reactive oxygen species (ROS) such as hydroxyl radicals (OH) and superoxide radicals (O_2_^−^) [11].

Peppers are susceptible to salt stress and about 14% of yield loss is due to salinity [11]. Soil is considered to be saline when the electric conductivity of the soil solution reaches 4 dS m^−1^ (equivalent to 40 mM NaCl). The salinity threshold level of pepper plants is 1.5 dS m^−1^, thus pepper is considered to be moderately salt-sensitive [7]. Plants can respond to salt stress by synthesizing secondary metabolites as one of the defense systems to reduce damage. The most commonly synthesized metabolites are flavonoids and phenolic acids [12]. Many studies have shown that salt stress and its effects on the plant are species- and crop-specific [13]. 

In our study, we examined four hydroponically grown hot pepper cultivars to see how they responded to two different salinity levels. By using hydroponics, we can control salinity so that it is easier to maintain a specific salinity level and expose plants to a desired stress intensity [14]. We analyzed the individual sugars, organic acids, phenolics, and capsaicinoids to determine how the pepper fruits responded to salt stress. We separated the fruit into three fruit parts (pericarp, placenta, and seed) to get a comprehensive picture of how each part of the fruit is affected by salinity. With this study, we captured a broad spectrum of metabolites that covers an important part of our knowledge on salt stress and the response of the species *Capsicum annuum* L. to it.

## 2. Materials and Methods

### 2.1. Experimental Design

A hydroponic experiment was conducted in Ljubljana (46°3′4″ N; 14°30′18″ E) at the Biotechnical Faculty from 20 May to 20 August 2020. A total of four pungent *Capsicum annuum* L. cultivars (‘Somborka’, ‘Novosadka’, ‘Berenyi F1’, and ‘Caro F1’) were sown in February. ‘Somborka’ and ‘Novosadka’ were purchased from NS seme and ‘Berenyi F1’ and ‘Caro F1’from Austrosaat. ‘Caro F1’ and ‘Somborka’ both form elongated, cone-shaped fruits that are quite large, and ‘Novosadka’ and ‘Berenyi F1’ form round, small fruits. All four cultivars were harvested at full size, in technological mature stage, as reported by Villaseñor et al. [15]. 

The seedlings were planted in March in 8 cm high plastic pots with peat substrate. When the first 4 to 5 true leaves appeared, the peat substrate was washed off the root system and the seedlings were placed in plastic hydroponic pots that were filled with mineral wool. The seedlings were watered constantly to allow the roots to overgrow the mineral wool substrate. On 20 May, the seedlings were placed in a closed pot drip hydroponic system (Wilma; Nutriculture, Lancashire, UK).

Each of the six 80 L systems (two systems for one treatment) consisted of 8 plastic pots (20 cm × 20 cm × 30 cm) that were standing on a plastic plate, each connected to a drip to supply water and minerals. The pots were filled with perlite substrate and each contained one plant (plants were randomly placed in rows 50 cm apart and 25 cm between the pots). The water and fertilizers were changed every two to three weeks, depending on the water consumption of the plant. The mineral solution was based on Kacjan Maršić et al. [16]. The pH and electrical conductivity (EC) of the water solution were, on average, 6.0 and 2.2 dS m^−1^, respectively.

A total of three treatments were performed: (1) control, (2) 20 mM NaCl, and (3) 40 mM NaCl. The NaCl (Sigma Aldrich) was added when the first fruits formed after flowering (salt stress was maintained for 10 weeks). Each time the water solution was changed, the systems were completely drained and the NaCl treatments were refilled with fresh NaCl salt. For the 20 mM NaCl treatment, 1168.75 mg L^−1^ NaCl was added and for the 40 mM NaCl treatment, 2337.50 mg L^−1^ NaCl was added. The pH and EC were 6.1 and 4.5 dS m^−1^ for the 20 mM NaCl treatment and 6.0 and 5.8 dS m^−1^ for the 40 mM treatment. Measurements were taken weekly using an electrical conductivity and pH meter (CyberScan PCD650; Eutech Instruments, Singapore) to monitor pH and EC. The pH was kept optimal for peppers, between 5.8 and 6.1, as previously reported by Kacjan Maršić et al. [16]. When the pH was not in the optimal range, 74% H_2_SO_4_ was added. The average temperature in the greenhouse was 23.1 °C and the relative humidity was 69.4%.

### 2.2. Fruit Sampling

Each of the three treatments was repeated twice (two systems for one treatment) so that each contained four plants of the same cultivar. Sampling was performed when the fruits reached a sufficient size, firmness, and color (yellow) [15]. Each plant (three to four fruits from each plant) represented a separate replicate. The samples were separated into pericarp, placenta, and seeds and freeze-dried. The dry samples were ground in a mortar and stored at −20 °C until further processing.

### 2.3. Extraction of Sugars and Organic Acids

Dry samples (0.05 g) were extracted with 3 mL of bidistilled water, for sugars and organic acids and shaken for 30 min on an orbital shaker (300 rpm). The samples were then centrifuged at 10,000× *g* for 5 min and filtered through a 0.25 µm cellulose filter (Chromafil A-20/25; Macherey-Nagel, Dueren, Germany) and stored in vials at −20 °C.

Ascorbic acid extraction was performed using 0.05 g of dry sample and 4 mL of 2% metaphosphoric acid. Further processing of the samples was the same as for the other organic acids. The HPLC system, columns, and mobile phases for the analysis of sugars and organic acids were the same as previously described by Zamljen et al. [17]. All the sugars and organic acids were expressed in g 100 g^−1^ dry weight (DW).

### 2.4. Extraction of Phenolics and Capsaicinoids

For the extraction of phenols and capsaicinoids, 0.05 g of dry sample was extracted with 2 mL of 80% methanol. The samples were then placed in a chilled ultrasonic bath for one hour and centrifuged at 8000× *g* for 5 min. They were filtered through a 0.25 µm polyamide filter (Chromafil AO-20/25, Macherey-Nagel, Dueren, Germany) and stored at −20 °C until analysis.

The identification of individual phenols was performed separately for all three fruit parts by tandem mass spectrometry (LTQ XL; Thermo Scientific, Waltham, MA, USA) with heated electrospray ionization in negative ion mode. The settings were the same as previously reported by Medic et al. [18]. The quantification of individual phenols was performed using a UHPLC system (Vanquish; Thermo Scientific, Waltham, MA, USA). The UHPLC system settings and column were the same as previously reported by Medic et al. [19]. The chromatographic data of individual phenolics for pericarp, placenta, and seeds are presented in Figures S1–S3.

The identification of capsaicinoids was performed using a tandem mass spectrometer (LTQ XL; Thermo Scientific, Waltham, MA, USA). The quantification of capsaicinoids was performed using a UHPLC-PDA Thermo Scientific Dionex UltiMate 3000 HPLC (Thermo Scientific) system combined with a TSQ Quantum Access Max Quadrupole mass spectrometer (MS) (Thermo Fischer Scientific Institute, Waltham, MA, USA). Full details of the settings, column, and mobile phases are described by Zamljen et al. [20]. The data for individual phenols and capsaicinoids were expressed as mg 100 g^−1^ DW. 

All phenols and capsaicinoids were calculated based on the appropriate standards. Where no standard was available, we calculated the data as equivalents of similar substances that were available as standards. All caffeic acid hexosides were calculated on the basis of caffeic acid. All ferulic acid hexosides were calculated on the basis of ferulic acid. All coumaroylquinic acid derivatives and *p*-coumaroylquinic acid were calculated on the basis of *p*-coumaric acid. Chlorogenic acid was calculated based on 5-caffeoylquinic acid. All apigenin hexosides were calculated on the basis of apigenin-7-glucoside. All luteolin hexosides and tricin were calculated on the basis of luteolin-7-glucoside and all kaempferol hexosides based on kaempferol-3-glucoside, and quercetin rutinoside to the standard of quercetin rutinoside and quercetin rhamnoside to quercetin rhamnoside. Isorhamnetin rhamnoside was calculated based on the isorhamnetin-3-glucoside standard. Homocapsaicin was expressed as capsaicin equivalent and homodihydrocapsaicin as dihydrocapsaicin equivalent.

### 2.5. Chemicals

Several standards were used for all individual primary and secondary metabolites. For the sugars sucrose, glucose, and fructose (Sigma–Aldrich Chemie GmbH, Steinheim, Germany); for organic acids oxalic acid, citric acid, malic acid, succinic acid, quinic acid, ascorbic acid, and fumaric acid (Sigma–Aldrich Chemie GmbH, Steinheim, Germany); for individual phenolics caffeic acid, *p*-coumaric acid, ferulic acid, 5-caffeoxlquinic acid, apigenin-7-glucoside, luteolin-7-glucoside, kaempferol-3-glucoside, quercetin rhamnoside, quercetin rutinoside, and isorhamnetin-3-glucoside (Sigma–Aldrich Chemie GmbH, Steinheim, Germany); and for capsaicnoids capsaicin, dihydrocapsaicin, and nordihydrocapsaicin (Sigma–Aldrich Chemie GmbH, Steinheim, Germany).

### 2.6. Statistical Analysis

The data were evaluated with the use of the R statistical environment. The data are expressed as means ± standard error (SE). For the determination of significant differences between the salinity treatments and control, one-way analysis of variance (ANOVA) was used, with Dunnett’s test (α < 0.05). Each cultivar and fruit part were analyzed separately. 

## 3. Results 

### 3.1. Individual and Total Sugars

The individual and total sugar content is shown in Table 1. We observed a significant decrease in the total sugar content in the 40 mM NaCl treatments. In the pericarp of ‘Somborka’, the total sugar content decreased by 2.58 g 100 g^−1^ DW when it was treated with 40 mM NaCl compared to the control. In the placenta of ‘Somborka’, 40 mM NaCl treatment decreased the glucose and fructose content by 2.25 g 100 g^−1^ DW and 2.21 g 100 g^−1^ DW, respectively, compared to the control treatment. In the cultivar ‘Novosadka’, treatment with 40 mM NaCl negatively affected the fructose content in pericarp and the glucose, fructose, and total sugar content in the placenta.

The cultivar ‘Berenyi F1’ had 2.38 g 100 g^−1^ DW lower glucose content when it was treated with 20 mM NaCl and 2.26 g 100 g^−1^ DW lower glucose content when it was treated with 40 mM NaCl, compared to the control. In the placenta, both saline treatments had a negative effect on the glucose and fructose content and, consequently, on the total sugar content. The sucrose content in the pericarp of ‘Caro F1’ was higher in the 40 mM NaCl treatment than in the control. In the placenta of ‘Caro F1’, both salinity treatments had a negative effect on the fructose content compared to the control.

### 3.2. Individual and Total Organic Acids

In salt-stressed plants of ‘Somborka’ (pericarp), the contents of citric acid and ascorbic acid increased by 0.25 g 100 g^−1^ DW and 0.76 g 100 g^−1^ DW, respectively, in 20 mM and 40 mM NaCl treatments (Table 2). The total acid contents increased in the pericarp in both NaCl treatments. In the placenta, the oxalic acid, citric acid, succinic acid, and ascorbic acid decreased in the NaCl treatments compared to the control. The total acid contents in the placenta of ‘Somborka’ decreased by 2.06 g 100 g^−1^ DW and 5.11 g 100 g^−1^ DW in the 20 mM and 40 mM NaCl treatment, respectively, compared to the control treatment. 

In ‘Novosadka’, succinic acid in the pericarp decreased in both the NaCl treatments. The total organic acid content also changed in both the NaCl treatments by 3.22 g 100 g^−1^ DW for the 20 mM and 3.31 g 100 g^−1^ DW for the 40 mM NaCl treatment. In the placenta of ‘Novosadka’, only malic acid was affected by salinity, with an increase of 0.31 g 100 g^−1^ DW for the 20 mM NaCl treatment and 0.67 g 100 g^−1^ DW for the 40 mM NaCl treatment.

In ‘Berenyi F1’, oxalic acid and fumaric acid in the pericarp decreased under salt stress. Salt stress in the pericarp of ‘Caro F1’ decreased the content of oxalic acid, succinic acid, and fumaric acid compared to the control. In the placenta of ‘Caro F1’, oxalic acid, malic acid, succinic acid, and fumaric acid decreased under salt stress. The total organic acid contents in the placenta of ‘Caro F1’ decreased the most under 20 mM NaCl, with a decrease of 5.98 g 100 g^−1^ DW compared to the control.

### 3.3. Individual and Total Analyzed Phenolics 

#### 3.3.1. Identification of Individual Phenolics

The most phenolics were identified in the pericarp (18) followed by the placenta (7) and seeds (8) (Table 3). The only two phenolics that were found in all three fruit parts were ferulic acid hexoside 1 and luteolin-8-C-hexoside 1. A total of eight hydroxycinnamic acids were identified: (i) caffeic acid derivatives were identified through the fragmentation patterns of MS^n^ *m*/*z* 179, as previously reported by Medic et al. [19]; (ii) ferulic acid hexosides were identified through the fragmentation pattern MS^n^ *m*/*z* 178, 149 and 134, as reported by Guclu et al. [21] and their typical loss of hexose (−162), as reported by Medic et al. [22]; (iii) chlorogenic acid was identified through its fragmentation pattern of MS *m*/*z* 353, MS^2^ *m*/*z* 293 and MS^3^ *m*/*z* 191, as reported by Hossain et al. [23]; and (iv) *p*-coumaroylquinic acid derivatives were identified through their fragmentation pattern MS^n^ *m*/*z* 163, as previously reported by Medic et al. [19].

A total of eight flavones were identified through their fragmentation patterns. (i) Apigenin pentosyl derivatives were identified through the fragmentation patterns MS^n^ *m*/*z* 353 and *m*/*z* 325, as previously reported by Guclu et al. [21]. (ii) Luteolin-8-C-hexosides were identified through the fragmentation pattern MS^n^ *m*/*z* 357 and *m*/*z* 209 and luteolin-7-*O*-hexosides through the fragmentation pattern MS^n^ *m*/*z* 357, as reported by Mikulic-Petkovsek et al. [24]. Tricin was identified through the fragmentation pattern MS *m*/*z* 329, MS^2^ *m*/*z* 314, as reported by Kang et al. [25] and was placenta specific.

A total of six flavanols were identified, of which five were in the pericarp. (i) Quercetin glycosides were identified through their fragmentation pattern MS^n^ *m*/*z* 301, 300 and 179 and the help of an external standard; (ii) Kaempferol glycosides were identified with the fragmentation pattern of MS^n^ *m*/*z* 257 and 241 and isoramnetin rhamnoside through the fragmentation pattern MS^n^ *m*/*z* 314 and 315, as previously reported by Medic et al. [19], Guclu et al. [21]. 

#### 3.3.2. Quantification of Individual Phenolics

**Figure 1:**
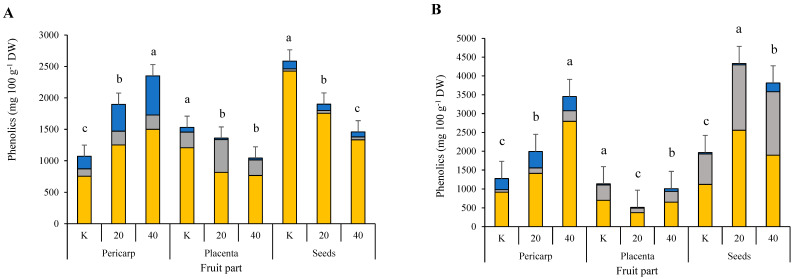

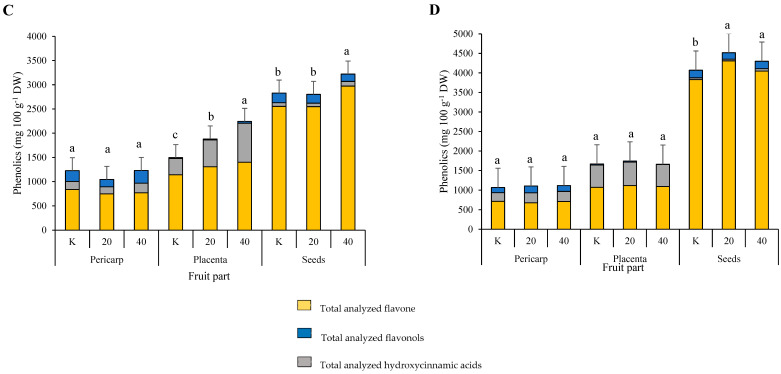
Total phenolics contents in four salt stressed pepper (*Capsicum annuum* L.) cultivars (‘Caro F1’ (**A**); ‘Somborka’ (**B**); ‘Novosadka’ (**C**); ‘Berenyi F1’ (**D**)) divided into three fruit parts. a, b, c indicates statistical differences among the NaCl treatments and the control. K = control; 20 = 20 mM NaCl; 40 = 40 mM NaCl.

The content of individual phenols in the pericarp of ‘Somborka’ was higher in both the NaCl treatments, with a few individual phenols being non-significant (Table S1). The total hydroxycinnamic acid content in the pericarp was 44.83 mg 100 g^−1^ DW and 204.01 mg 100 g^−1^ DW higher in the 20 mM and 40 mM NaCl treatments than in the control. The analyzed total flavone content of the pericarp was 495.75 mg 100 g^−1^ DW and 879.04 mg 100 g^−1^ DW higher in the 20 mM and 40 mM NaCl treatments than in the control. The total analyzed flavanols were also higher in the salt stressed plants. The total phenols analyzed in the pericarp of ‘Somborka’ were higher by 708.80 mg 100 g^−1^ DW in the 20 mM NaCl treatment and 1161.06 mg 100 g^−1^ DW in the 40 mM NaCl treatment than in the control. In the placenta of ‘Somborka’, we observed a negative effect of salinity on the content of phenols. Total hydroxycinnamic acids and total analyzed flavones were lower in the salt stressed treatments. Consequently, the total content of phenols that were analyzed was lower in the treatments with salt stress than in the control (Figure 1). Similar to the pericarp and placenta, the seeds of ‘Somborka’ also responded similarly to salt stress.

The pericarp of ‘Novosadka’ was less affected by the NaCl treatments, with only one effect on a single phenol, ferulic acid hexoside 1, which increased by 1.43 mg 100 g^−1^ DW and 9.18 mg 100 g^−1^ DW when treated with 20 mM and 40 mM NaCl, respectively, compared to the control (Table S2). In the placenta of ‘Novosadka’, the total analyzed hydroxycinnamic acids and flavones rose in both NaCl treatments. The total phenols that were analyzed in placenta increased by 341.43 mg 100 g^−1^ DW and 683.78 mg 100 g^−1^ DW for the 20 mM and 40 mM NaCl treatments, respectively, compared to the control. In the seeds of ‘Novosadka’, the total hydroxycinnamic acids and flavones that were analyzed increased, although the total flavones that were analyzed did not increase in the 20 mM NaCl treatment, but only in the 40 mM NaCl treatment compared to the control. The total phenols that were analyzed in the seeds of ‘Novosadka’ were higher (393.29 mg 100 g^−1^ DW higher) in the 40 mM NaCl treatment. The 20 mM NaCl treatment had no significant effect on the total phenolic content that was analyzed in the seeds of ‘Novosadka’ compared to the control treatment (Figure 1).

In the pericarp of ‘Berenyi F1’ (Table S3), the 40 mM NaCl treatment increased the total content of hydroxycinnamic acids by 28.47 mg 100 g^−1^ DW compared to the control treatment. The treatment with 20 mM NaCl had no effect on the total hydroxycinnamic acids that were analyzed compared to the control. Luteolin-8-C-hexoside 1 decreased and luteolin-7-*O*-hexoside increased in both NaCl treatments. Isorhamnetin rhamnoside increased in both NaCl treatments compared with the control. In the placenta of cultivar ‘Berenyi F1’, salt stress had no effect on the phenolic content. In the seeds, the 40 mM NaCl treatment had a significant effect on the total hydroxycinnamic acids that were analyzed compared to the control. On the other hand, the total flavones that were analyzed were more affected by the 20 mM NaCl treatment, with a content of 478.90 mg 100 g^−1^ DW higher than the control. When considering the total phenols that were analyzed, both NaCl treatments had a significant effect compared to the control (Figure 1).

The content of the analyzed hydroxycinnamic acids in the pericarp of ‘Caro F1’ increased in both NaCl treatments. Both salt stress treatments had a significant effect on individual phenols from the hydroxycinnamic acids group, with the exception of caffeic acid hexoside 2 (Table S4). The total flavones that were analyzed increased by 496.78 mg 100 g^−1^ DW in the 20 mM NaCl treatment and by 745.80 mg 100 g^−1^ DW in the 40 mM NaCl treatment compared to the control. The total flavonols that were analyzed increased in both salt stress treatments and, consequently, the individual flavonols also increased. The total phenols that were analyzed in the pericarp of cultivar ‘Caro F1’ increased by 829.25 mg 100 g^−1^ DW and 1275.18 mg 100 g^−1^ DW in the 20 mM and 40 mM NaCl treatments, respectively, compared to the control treatment (Figure 1). In the placenta of ‘Caro F1’, total hydroxycinnamic acids increased in the 20 mM NaCl treatment but not in the 40 mM NaCl treatment. The flavones and flavonols in the placenta were negatively affected by the salt stress treatments, as were the total phenolics that were analyzed. Similar to the placenta, the seeds also showed lower levels of phenols in the salt stress treatments.

#### 3.3.3. Individual and Total Capsaicinoids

**Figure 2:**
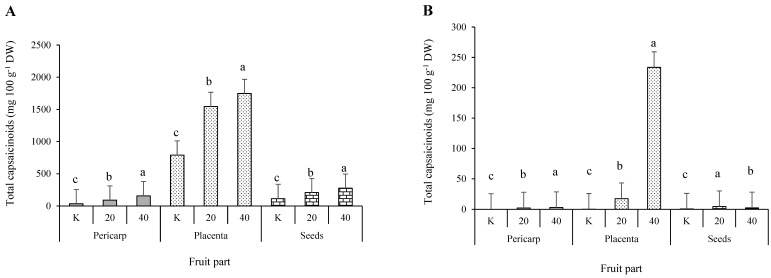

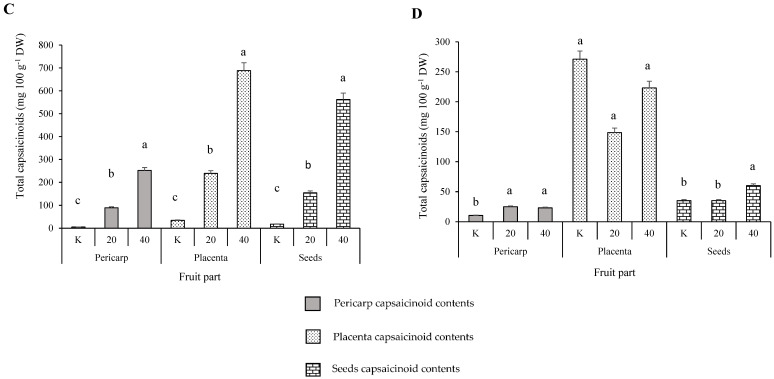
The total analyzed capsaicinoids contents in four salt stressed pepper (*Capsicum annuum* L.) cultivars (‘Caro F1’ (**A**); ‘Somborka’ (**B**); ‘Novosadka’ (**C**); ‘Berenyi F1’ (**D**)), divided into three fruit parts. a, b, c indicates statistical differences among the NaCl treatments and control. K = control; 20 = 20 mM NaCl; 40 = 40 mM NaCl.

Individual capsaicinoids in the pericarp of the cultivar ‘Somborka’ (Table S5) were higher in the salt stressed treatments. Nordihydrocapsaicin, homocapsaicin, and homodihydrocapsaicin were not detected. Total capsaicinoid content (Figure 2) in the pericarp was 2.12 mg 100 g^−1^ DW and 2.96 mg 100 g^−1^ DW higher in the 20 mM and 40 mM NaCl treatments than in the control treatment, respectively. In the placenta, the increase in all five individual capsaicinoids was high in the 20 mM NaCl treatment and very high in the 40 mM NaCl treatment. The total increase of capsaicinoids in the placenta with the 20 mM NaCl treatment was 17.38 mg 100 g^−1^ DW and with the 40 mM NaCl treatment 233.32 mg 100 g^−1^ DW compared to the control. No homodihydrocapsaicin was detected in the seeds of ‘Somborka’. The other four capsaicinoids increased under salt stress. The total capsaicinoid content in the seeds was highest in the 20 mM NaCl treatment, followed by the 40 mM NaCl treatment.

In ‘Novosadka’, all three fruit parts responded similarly to salinity (Figure 2). All five identified capsaicinoids increased with the 20 mM NaCl and 40 mM NaCl treatments, and so did the total amount of capsaicinoids (Table S5) in all fruit parts.

The pericarp of cultivar ‘Berenyi F1’ was affected by 20 mM and 40 mM NaCl treatments in terms of individual capsaicinoids. The total capsaicinoid content increased by 14.59 mg 100 g^−1^ DW in the 20 mM NaCl treatment and by 12.62 mg 100 g^−1^ DW in the 40 mM NaCl treatment as compared to the control treatment. In the placenta of ‘Berenyi F1’, no differences were observed between the treatments. In the seeds, the capsaicin content increased by 15.03 mg 100 g^−1^ DW and the dihydrocapsaicin content increased by 6.25 mg 100 g^−1^ DW in the 40 mM NaCl treatment compared to the control treatment. The total capsaicinoid content in the seeds increased by 24.86 mg 100 g^−1^ DW in the 40 mM NaCl treatment compared to the control.

‘Caro F1’ responded to salt stress with an increased accumulation of individual and total capsaicinoids in the pericarp, placenta, and seeds (Table S5). The 20 mM NaCl treatment showed a high increase and the 40 mM NaCl treatment showed a very high increase in capsaicinoids compared to the control. The total capsaicinoid content in the pericarp of peppers that were treated with 20 mM NaCl was 2.6 times higher than the control and in the 40 mM NaCl treatment was 4.5 times higher than the control. In the placenta, the increase was 1.9-fold and 2.2-fold for the 20 mM and 40 mM NaCl treatments, respectively. In the seeds, the increase in total capsaicinoids was 1.8-fold and 2.4-fold in the 20 mM and 40 mM NaCl treatments, respectively, compared to the control treatment (Figure 2).

## 4. Discussion

A total of four pepper cultivars were tested in a hydroponic trial to investigate the effects of salinity or salt stress on pepper fruit quality. For a more comprehensive analysis, the fruits were divided into three fruit parts consisting of the pericarp, placenta, and seed. We observed an effect of salinity, genotype, and fruit part in all four cultivars. The individual and total sugars decreased significantly in all salinity treatments, except for the sucrose content of the pericarp of ‘Caro F1’, with salinity having the greatest effect on the glucose and fructose. The content of organic acids increased in the pericarp of ‘Somborka’ and decreased in all other treatments. The most affected organic acids were citric acid, succinic acid, fumaric acid, and ascorbic acid. As for the individual phenols, most of them increased with increasing salinity, although there were some that decreased, such as luteolin-8-C-hexoside 1. Interestingly, the total phenols in the placentas of ‘Caro F1’ and ‘Somborka’ decreased in the salinity treatments and increased in the pericarps. Capsaicinoid content increased under salt stress, with a medium increase in the 20 mM NaCl treatment and a high increase in the 40 mM NaCl treatment in all the cultivars and fruit parts. 

The plants that were exposed to salt stress have several mechanisms to reduce the negative effects, such as ion homeostasis, compatible solutes, also known as compatible osmolytes (proline, sugars, glycine betaine, and polyols), antioxidant regulation, polyamines, and hormones [16]. In ‘Somborka’ and ‘Novosadka’, neither the sugar contents changed compared to the control when exposed to medium stress with the 20 mM NaCl treatment but increased at 40 mM NaCl stress. On the other hand, both hybrid cultivars also showed lower sugar contents under medium stress with the 20 mM NaCl treatment and with the 40 mM NaCl treatment. Our results are similar to those of Saied et al. [26], who reported a decrease in glucose and fructose in strawberry due to salinity. The reason for the lower sugar content in peppers might be that high salinity leads to an accumulation of ions (especially Cl^−^) in the plant, especially in the leaves, thereby reducing the photosynthetic rate and CO_2_ fixation [26]. With a lower photosynthetic rate, electron transfer in the photosystem II decreases, affecting sugar accumulation, distribution, and translocation in plant tissues, as reported by Lopez et al. [27], which may be the reason for the lower sugar levels under salt stress. Similarly, to the sugars, organic acids also decreased, except in the pericarp of ‘Somborka’, in which we observed an increase. Organic acids and sugars are closely related since organic acids are the result of incomplete oxidation of photosynthetic assimilates [28]. They can be converted back to sugars or oxidized to CO_2_ and H_2_O. Their carbon skeletons can be used for the biosynthesis of amino acids, which are important for the further synthesis of secondary metabolites. Organic acids have an important function in plants in maintaining redox balance, production, and consumption of ATP, and supporting proton and ion gradients at the membranes [28]. In plants that are subjected to salt stress or other abiotic stress, reactive oxygen species (ROS) rapidly form and disrupt the balance between the production and degradation of ROS. With increased ROS production, macromolecules such as nucleic acids, proteins, carbohydrates, and lipids are damaged and lead to cell death [29], which could explain the lower sugar content in our study. The decrease in sugars and organic acids under salt stress could indicate that these primary metabolites are used for the synthesis of secondary metabolites, such as phenols and capsaicinoids. 

We observed different responses of cultivars and fruit parts to salinity with respect to individual and total phenols. Phenols are defense molecules that are synthesized under stress conditions such as salt stress [29]. As mentioned earlier, salt stress increases the formation of superoxide anions, hydrogen peroxide, and hydroxyl ions [30]. Phenols are potent antioxidants in plants and help to reduce the negative effects of ROS in cells that are caused by salt stress. Salt stress stimulates gene expression for increased activity of the phenylpropanoid biosynthetic pathway to produce various phenolic compounds that have potent antioxidant activity [31]. As reported by Golkar et al. [32], several genes are involved in increased production of flavonoids by regulating biosynthetic pathways and conferring salt tolerance to plants. In *Glycine max*, the flavone synthase genes were up regulated under salt stress [33]. As previously reported by Ben et al. [34] and Scagel et al. [35] phenolic acids such as caffeic acid, coumaroylquinic acid, ferulic acid, and chlorogenic acid accumulate in plants under salt stress, which we also confirmed in the *Capsicum annuum* cultivars that were used in our study. 

The greatest effect and response to salt stress was observed in the levels of individual and total capsaicinoids. We observed a significant increase in both salt treatments. The content of capsaicinoids increased with increasing salt stress, as also previously reported by Arrowsmith et al. [36] and Julien et al. [37]. We also observed that ‘Berenyi F1’ was affected in the pericarp only by the highest salinity of 40 mM and not by 20 mM NaCl. This might indicate that it is more resistant to low intensity salt stress. In the other three cultivars, we observed a significant increase in capsaicinoids with increasing salt stress. As mentioned earlier, salt stress affects the phenylpropanoid biosynthetic pathway and stimulates its activity. The products of phenylalanine ammonia lyase (PAL enzyme) activity are important for further phenol synthesis and hence capsaicinoids. The PAL enzyme activity and capsaicinoid content are closely related, as previously reported by Castro-Concha et al. [38], which could explain our results. 

Our study showed that genotype is very important for the metabolic response to salt stress. We observed very different metabolite statuses among the four cultivars, which was previously reported in peppers by Shams and Yildirim [39] in three genotypes of peppers and in 102 genotypes of peppers by Aktas et al. [40]. As mentioned earlier, ROS is produced in large amounts in salt stressed plants. Peppers under salt stress rely on metabolic processes, signaling molecules, and hormones [41]. Nutrient and water uptake is poor under salt stress, which leads to a reduction in growth and also metabolic processes in plants. Poor uptake of water and nutrients is also affected by salt-induced osmotic stress [42]. In salt stressed plants, the intensity of cell division and elongation are lower [39] and all these processes are controlled by genotype and genes.

## 5. Conclusions

We examined the response of four *C. annuum* genotypes to two intensities of salt stress. We analyzed all three fruit parts of each cultivar to obtain a more detailed picture of the effects of salt stress. We found that the metabolic response depended on the cultivar, fruit part, and salinity level. In most cases, the response of phenolics, and especially capsaicinoids, increased with the intensity of salt stress. In general, the sugar content decreased with few exceptions, and salt stress had a particular effect on glucose and fructose. Organic acids also decreased in most samples, with the exception of the pericarp of ‘Somborka’. With the results of this study, we performed a comprehensive analysis that fills a gap in our knowledge of how primary and secondary metabolites respond to salt stress in pepper plants, which has rarely been done. With the metabolic results of this study, we have laid the foundation for further studies, which would concentrate on the enzymatic and genomic reactions that are associated with salt stress in hot peppers. 

## Figures and Tables

**Table 1:** Individual and total sugar contents (g 100 g^−1^ DW; mean ± SE) of three fruit parts of the four pepper (*Capsicum annuum* L.) cultivars under salt stress.

Cultivar/Fruit Part		Sugar	Treatment						
			Control		20 mM NaCl		40 mM NaCl		Significance
**‘Somborka’**	Pericarp	Sucrose	5.53 ± 0.26	a	5.22 ± 0.25	a	4.96 ± 0.15	a	NS
		Glucose	7.13 ± 0.16	a	7.36 ± 0.63	a	6.02 ± 0.27	a	NS
		Fructose	6.77 ± 0.12	a	6.77 ± 0.51	a	5.88 ± 0.26	a	NS
		**Total sugars**	**19.44 ± 0.55**	**a**	**19.35 ± 1.39**	**a**	**16.86 ± 0.69**	**b**	**
	Placenta	Sucrose	3.74 ± 0.55	a	4.85 ± 0.10	a	5.00 ± 0.17	a	NS
		Glucose	5.07 ± 0.22	a	5.53 ± 0.12	a	2.82 ± 0.19	b	***
		Fructose	5.69 ± 0.24	a	5.48 ± 0.84	a	3.48 ± 0.22	b	***
		**Total sugars**	**14.51 ± 1.01**	**a**	**15.87 ± 3.15**	**a**	**11.31 ± 0.54**	**a**	NS
**‘Novosadka’**	Pericarp	Sucrose	4.50 ± 0.67	a	6.35 ± 0.14	a	5.56 ± 0.91	a	NS
		Glucose	10.44 ± 0.84	a	9.30 ± 0.40	a	8.60 ± 081	a	NS
		Fructose	9.14 ± 0.61	a	8.42 ± 0.41	ab	7.19 ± 0.34	b	**
		**Total sugars**	**24.09 ± 2.12**	**a**	**24.08 ± 2.26**	**a**	**21.37 ± 2.07**	**a**	NS
	Placenta	Sucrose	9.12 ± 0.64	a	9.45 ± 0.21	a	8.36 ± 0.14	a	NS
		Glucose	5.23 ± 0.22	a	4.51 ± 0.32	a	3.39 ± 0.19	b	***
		Fructose	4.92 ± 0.17	a	4.44 ± 0.35	a	3.17 ± 0.08	b	**
		**Total sugars**	**19.28 ± 1.04**	**a**	**18.42 ± 2.79**	**a**	**14.92 ± 0.42**	**b**	***
**‘Berenyi F1’**	Pericarp	Sucrose	3.93 ± 0.28	a	3.73 ± 0.24	a	3.60 ± 0.24	a	NS
		Glucose	9.96 ± 0.21	a	7.57 ± 1.23	b	7.69 ± 1.53	b	***
		Fructose	9.08 ± 0.16	a	9.65 ± 1.61	a	7.22 ± 1.41	a	NS
		**Total sugars**	**22.97 ± 0.68**	**a**	**20.96 ± 3.09**	**a**	**18.52 ± 3.18**	**a**	NS
	Placenta	Sucrose	6.82 ± 0.86	a	6.92 ± 0.91	a	6.97 ± 0.26	a	NS
		Glucose	4.97 ± 0.48	a	3.87 ± 0.31	b	3.54 ± 0.21	b	***
		Fructose	6.05 ± 1.54	a	3.77 ± 0.24	b	3.56 ± 0.24	b	***
		**Total sugars**	**17.85 ± 2.90**	**a**	**14.56 ± 1.47**	**b**	**14.07 ± 0.72**	**b**	***
**‘Caro F1’**	Pericarp	Sucrose	3.98 ± 0.69	b	6.07 ± 0.76	b	9.95 ± 0.77	a	***
		Glucose	9.42 ± 0.77	a	10.48 ± 0.11	a	9.36 ± 0.43	a	NS
		Fructose	8.35 ± 0.71	a	9.44 ± 0.21	a	7.64 ± 0.49	a	NS
		**Total sugars**	**21.75 ± 2.18**	**a**	**25.99 ± 1.08**	**a**	**26.95 ± 1.70**	**a**	NS
	Placenta	Sucrose	1.13 ± 0.21	a	1.09 ± 0.09	a	1.21 ± 0.19	a	NS
		Glucose	5.32 ± 0.31	a	4.41 ± 0.40	a	4.62 ± 0.14	a	NS
		Fructose	4.56 ± 0.33	a	3.76 ± 0.23	b	3.63 ± 0.04	b	***
		**Total sugars**	**11.01 ± 1.64**	**a**	**9.26 ± 1.73**	**a**	**9.46 ± 1.28**	**a**	NS

Different letters in the row (a, b) indicate statistical differences among the treatments. Significance codes: *** ≤ 0.001; ** ≤ 0.01; * ≤ 0.05; NS > 0.05.

**Table 2:** Individual and total organic acid contents (g 100 g^−1^ DW mean ± SE) of three fruit parts of the four pepper (*Capsicum annuum* L.) cultivars under salt stress.

Cultivar/Fruit Part		Organic Acid	Treatment						
			Control		20 mM NaCl		40 mM NaCl		Significance
**‘Somborka’**	Pericarp	Oxalic a.	0.22 ± 0.01	a	0.17 ± 0.02	a	0.18 ± 0.01	a	NS
		Citric a.	0.52 ± 0.02	b	0.77 ± 0.29	a	1.28 ± 0.10	a	***
		Malic a.	1.75 ± 0.09	a	1.84 ± 0.30	a	1.55 ± 0.08	a	NS
		Qunic a.	0.43 ± 0.05	a	0.84 ± 0.17	a	0.53 ± 0.10	a	NS
		Succinic a.	10.31 ± 0.14	a	10.51 ± 0.48	a	10.43 ± 0.01	a	NS
		Fumaric a.	0.021 ± 0.001	a	0.022 ± 0.002	a	0.017 ± 0.001	a	NS
		Ascorbic a.	3.06 ± 0.24	b	4.08 ± 0.29	a	4.72 ± 0.31	a	***
		**Total acids**	**16.33 ± 0.57**	**b**	**18.25 ± 0.15**	**a**	**18.72 ± 0.63**	**a**	***
	Placenta	Oxalic a.	0.35 ± 0.02	a	0.06 ± 0.06	b	0.17 ± 0.07	b	**
		Citric a.	1.11 ± 0.26	a	0.62 ± 0.07	b	0.68 ± 0.13	b	***
		Malic a.	2.12 ± 0.14	a	2.25 ± 0.21	a	1.61 ± 0.28	a	NS
		Qunic a.	0.08 ± 0.13	a	0.37 ± 0.26	a	0.06 ± 0.26	a	NS
		Succinic a.	14.79 ± 0.26	a	12.98 ± 0.78	b	10.68 ± 1.07	b	***
		Fumaric a.	0.023 ± 0.005	a	0.014 ± 0.003	a	0.012 ± 0.005	a	NS
		Ascorbic a.	0.11 ± 0.01	a	0.06 ± 0.01	b	0.04 ± 0.01	b	***
		**Total acids**	**18.39 ± 0.88**	**a**	**16.33 ± 1.45**	**b**	**13.28 ± 1.84**	**b**	***
**‘Novosadka’**	Pericarp	Oxalic a.	0.24 ± 0.02	a	0.20 ± 0.04	a	0.18 ± 0.01	a	NS
		Citric a.	0.89 ± 0.23	a	0.76 ± 0.21	a	1.05 ± 0.17	a	NS
		Malic a.	1.53 ± 0.29	a	1.68 ± 0.14	a	1.70 ± 0.12	a	NS
		Qunic a.	2.02 ± 1.31	a	0.84 ± 0.15	a	0.86 ± 0.15	a	NS
		Succinic a.	11.90 ± 0.48	a	10.92 ± 0.11	b	10.22 ± 0.20	b	***
		Fumaric a.	0.028 ± 0.005	a	0.025 ± 0.005	a	0.023 ± 0.001	a	NS
		Ascorbic a.	6.34 ± 0.92	a	5.29 ± 0.80	a	5.61 ± 0.19	a	NS
		**Total acids**	**22.97 ± 0.32**	**a**	**19.75 ± 1.47**	**b**	**19.66 ± 0.87**	**b**	***
	Placenta	Oxalic a.	0.40 ± 0.07	a	0.35 ± 0.01	a	0.23 ± 0.01	a	NS
		Citric a.	1.33 ± 0.08	a	1.20 ± 0.27	a	1.06 ± 0.09	a	NS
		Malic a.	1.40 ± 0.17	b	1.71 ± 0.21	a	2.07 ± 0.14	a	**
		Qunic a.	N/D		N/D		N/D		
		Succinic a.	10.93 ± 0.59	a	11.48 ± 0.66	a	10.46 ± 0.35	a	NS
		Fumaric a.	0.023 ± 0.005	a	0.030 ± 0.011	a	0.022 ± 0.003	a	NS
		Ascorbic a.	0.41 ± 0.06	a	0.35 ± 0.04	a	0.54 ± 0.12	a	NS
		**Total acids**	**14.49 ± 1.19**	**a**	**14.92 ± 1.38**	a	**14.41 ± 0.89**	a	NS
**‘Berenyi F1’**	Pericarp	Oxalic a.	0.16 ± 0.02	a	0.10 ± 0.01	b	0.09 ± 0.01	b	***
		Citric a.	1.62 ± 0.08	a	1.01 ± 0.04	a	1.56 ± 0.20	a	NS
		Malic a.	1.04 ± 0.01	a	1.27 ± 0.16	a	1.34 ± 0.23	a	NS
		Qunic a.	0.87 ± 0.04	a	0.73 ± 0.16	a	0.67 ± 0.17	a	NS
		Succinic a.	11.79 ± 0.28	a	10.45 ± 0.27	a	9.98 ± 0.81	a	NS
		Fumaric a.	0.046 ± 0.008	a	0.022 ± 0.001	b	0.014 ± 0.001	b	***
		Ascorbic a.	5.93 ± 0.55	a	5.13 ± 0.89	a	4.48 ± 1.43	a	NS
		**Total acids**	**21.47 ± 1.00**	**a**	**18.73 ± 1.55**	a	**18.17 ± 2.87**	a	NS
	Placenta	Oxalic a.	0.26 ± 0.01	a	0.22 ± 0.02	a	0.48 ± 0.22	a	NS
		Citric a.	1.58 ± 0.11	a	1.08 ± 0.10	a	1.53 ± 0.18	a	NS
		Malic a.	1.80 ± 0.04	a	1.64 ± 0.14	a	1.58 ± 0.20	a	NS
		Qunic a.	0.03 ± 0.00	a	0.15 ± 0.07	a	0.18 ± 0.10	a	NS
		Succinic a.	11.62 ± 0.08	a	11.61 ± 0.32	a	12.15 ± 0.32	a	NS
		Fumaric a.	0.022 ± 0.002	a	0.028 ± 0.005	a	0.022 ± 0.002	a	NS
		Ascorbic a.	0.39 ± 0.11	a	0.29 ± 0.06	a	0.30 ± 0.04	a	NS
		**Total acids**	**15.73 ± 0.52**	**a**	**15.04 ± 0.74**	**a**	**16.26 ± 1.09**	**a**	**NS**
**‘Caro F1’**	Pericarp	Oxalic a.	0.16 ± 0.02	a	0.09 ± 0.01	b	0.04 ± 0.01	b	***
		Citric a.	1.54 ± 0.23	a	1.52 ± 0.15	a	2.08 ± 0.10	a	NS
		Malic a.	1.62 ± 0.52	a	1.31 ± 0.21	a	0.78 ± 0.12	a	NS
		Qunic a.	1.76 ± 0.61	a	0.66 ± 0.17	a	0.99 ± 0.30	a	NS
		Succinic a.	11.51 ± 0.17	a	9.71 ± 0.01	b	10.45 ± 0.04	b	***
		Fumaric a.	0.033 ± 0.007	a	0.013 ± 0.001	b	0.012 ± 0.003	b	**
		Ascorbic a.	5.66 ± 0.97	a	6.12 ± 0.79	a	4.40 ± 0.77	a	NS
		**Total acids**	**22.30 ± 2.55**	a	**19.44 ± 1.37**	a	**18.77 ± 1.36**	a	NS
	Placenta	Oxalic a.	0.34 ± 0.01	a	0.15 ± 0.08	b	0.15 ± 0.01	b	***
		Citric a.	1.41 ± 0.03	a	1.24 ± 0.36	a	2.22 ± 0.48	a	NS
		Malic a.	3.96 ± 0.09	a	2.17 ± 0.83	b	2.00 ± 0.23	b	***
		Qunic a.	0.04 ± 0.02	a	0.28 ± 0.08	a	0.95 ± 0.29	a	NS
		Succinic a.	14.38 ± 0.37	a	10.34 ± 0.175	b	11.89 ± 0.53	b	**
		Fumaric a.	0.063 ± 0.01	a	0.025 ± 0.012	b	0.034 ± 0.004	b	***
		Ascorbic a.	0.54 ± 0.20	a	0.53 ± 0.14	a	0.42 ± 0.17	a	NS
		**Total acids**	**20.75 ± 0.73**	**a**	**14.77 ± 3.27**	**b**	**17.70 ± 1.74**	**b**	***

Different letters in the row (a, b) indicate statistical differences among the treatments. Significance codes: *** ≤ 0.001; ** ≤ 0.01; * ≤ 0.05; NS *>* 0.05.

**Table 3:** The tentative identification of the 22 phenolics from three fruit parts of peppers (*Capsicum annuum* L.).

Compound	Rt	[M-H]^−^	MS^2^	MS^3^	MS^4^	Plant Tissue		
	(min)	(*m*/*z*)	(*m*/*z*)	(*m*/*z*)	(*m*/*z*)	Pericarp	Placenta	Seeds
Coumaroylquinic acid derivative 1	7.39	391	216 (100), 173 (45), 111 (44), 191 (30), 129 (3)			×	×	
Tricin	9.27	329	314 (100), 311 (43), 285 (17)				×	
Caffeic acid hexoside 1	10.12	341	179 (100), 161 (26), 135 (4)			×		
Caffeic acid hexoside 2	11.95	341	179 (100), 135 (4)			×		×
*p*-Coumaroylquinic acid	12.18	371	**325** (100), 307 (80), 191 (61), 163 (46)	163 (100)		×		
Caffeic acid hexoside derivative	13.79	387	179 (100), 341 (69)			×	×	
Ferulic acid hexoside 1	14.07	355	**193** (100), 217 (55), 175 (30)	134 (100), 149 (52), 178 (22)		×	×	×
Ferulic acid hexoside 2	14.45	551	**389** (100), 193 (43), 341 (15)	341 (100), **193** (43)	149 (100), 178 (57), 134 (30)	×		×
Chlorogenic acid	15.35	353	**293** (100)	191 (100), 131 (55)		×		
Apigenin pentosyl hexoside 1	15.60	696	**469** (100), 353 (40), 243 (30)	325 (100), 353 (93), 243 (30)		×	×	
Caffeic acid hexoside 3	16.37	583	241 (100), **341** (40), 179 (1)	179 (100), 161 (23)				×
Apigenin pentosyl hexoside 2	17.03	563	**443** (100), 473 (86), 383 (20), 353 (19)	353 (100), 383 (14)		×		
Apigenin pentosyl hexoside 3	18.16	563	**443** (100), 473 (53), 353 (13)	**353** (100), 383 (35)	325 (100), 297 (30)	×		
Luteolin-8-C-hexoside 1	18.79	567	357 (100)	209 (100)		×	×	×
Luteolin-8-C-hexoside 2	20.77	567	477 (100), **447** (96), 387 (88), 357 (79)	**357** (100), 387 (74)	209 (100)	×		×
Kaempferol dihexoside	21.16	427	**397** (100), 257 (48), 241 (4)	257 (100), **241** (8)	97 (100), 231 (41), 151 (20)	×		
Quercetin rutinoside	22.19	609	301 (100), 300 (11), 179 (2)			×		
Quercetin rhamnoside	22.88	447	301 (100), 300 (26), 179 (1)			×	×	
Kaempferol hexoside 1	22.99	349	241 (100), 151 (10)					×
Luteolin-7-*O*- hexoside	23.05	665	**621** (100)	489 (100), 285 (56)		×		
Isorhamnetin rhamnoside	24.76	461	**314** (100), 315 (58)	285 (100), 286 (67), 271 (66), 243 (20)		×		
Kaempferol hexoside 2	25.43	393	241 (100), 349 (61), 257 (49)					×

Rt = retention time; [M-H]^−^ = main mass of substance in negative ion mode; MS^2^ = first fragmentation; MS^3^ = second fragmentation; MS^4^ = third fragmentation.

## Data Availability

The data presented in this study are available on request from the corresponding author. The data are not publicly available due to privacy.

## Supplementary Materials

The following supporting information can be downloaded at: https://www.mdpi.com/article/10.3390/plants11070853/s1, Figure S1: Full scan on a HPLC-MS, and the phenolic compounds that were identified for pericarp; Figure S2: Full scan on a HPLC-MS, and the phenolic compounds that were identified for placenta. Figure S3: Full scan on a HPLC-MS, and the phenolic compounds that were identified for seeds. Table S1: Individual and total phenolics of three fruit parts of the ‘Somborka’ cultivar under salt stress (mg 100 g^−1^ DW; mean ± SE). Table S2: Individual and total phenolics of three fruit parts of the ‘Novosadka’ cultivar under salt stress (mg 100 g^−1^ DW; mean ± SE). Table S3: Individual and total phenolics of three fruit parts of the ‘Berenyi F1’ cultivar under salt stress (mg 100 g^−1^ DW; mean ± SE). Table S4: Individual and total phenolics of three fruit parts of the ‘Caro F1’ cultivar under salt stress (mg 100 g^−1^ DW; mean ± SE). Table S5: Individual and total capsaicinoids contents of three fruit parts of the four cultivars under salt stress (mg 100 g^−1^ DW; mean ± SE).
