# Supplementary material for: Salt Stress Differentially Affects the Primary and Secondary Metabolism of Peppers (Capsicum annuum L.) According to the Genotype, Fruit Part, and Salinity Level
Source: Plants (Basel). 2022 Mar 23;11(7):853. doi: 10.3390/plants11070853 (PMC9003448; doi:10.3390/plants11070853)
Supplement: Supplementary file 1 [file plants-11-00853-s001.zip › plants-1630530-supplementary.pdf]

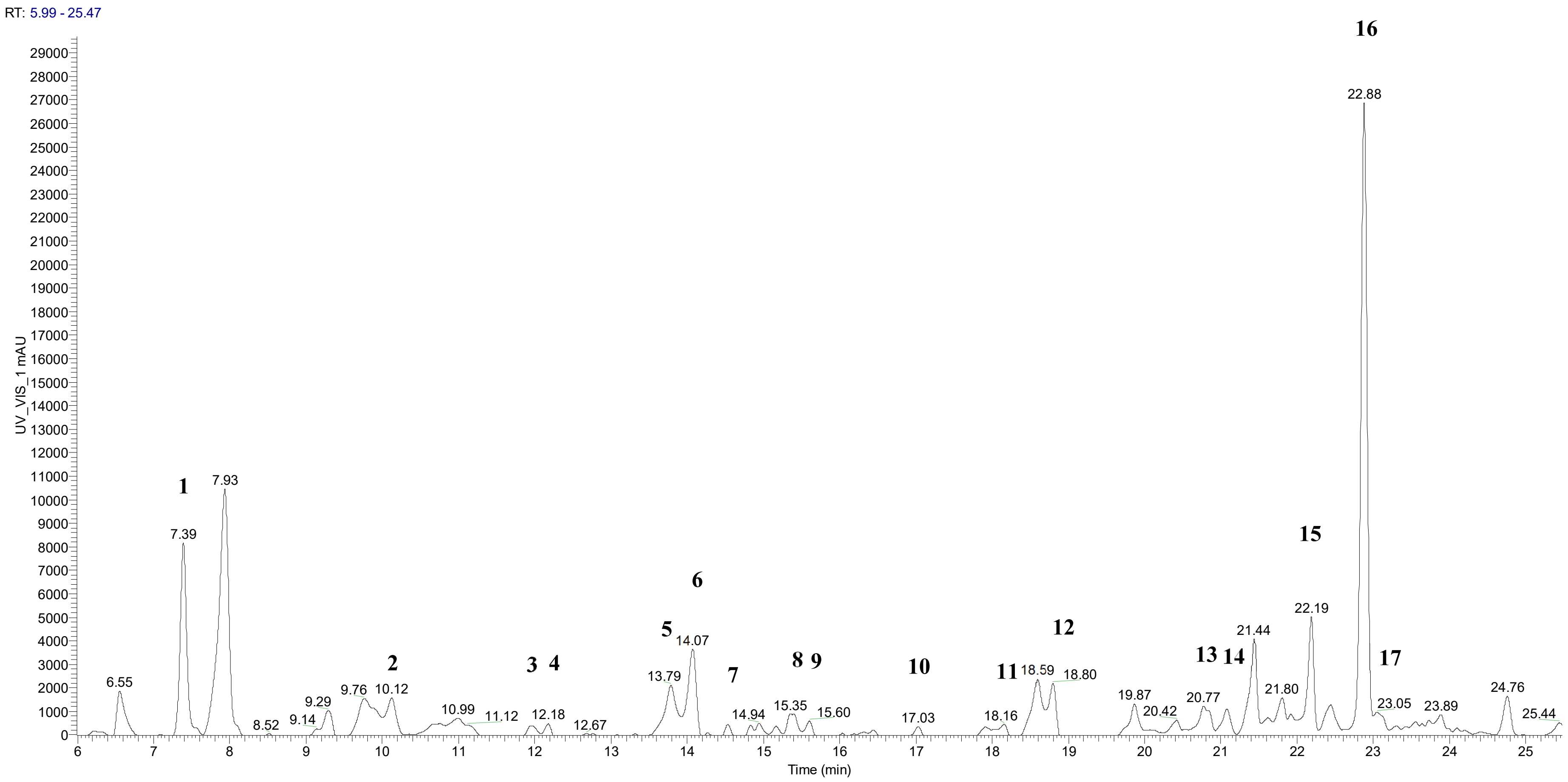

Figure S1. Full scan on a HPLC-MS, and the phenolic compounds identified for pericarp. **1**= Coumaroylquinic acid derivative 1; **2**= Caffeic acid hexoside 1; **3**= Caffeic acid hexoside 2; **4**= *p*-coumaroylquinic acid; **5**= Caffeic acid hexoside derivative; **6=** Ferulic acid hexoside 1; **7**= Ferulic acid hexoside 2; **8**= Chlorogenic acid; **9**= Apigenin pentosyl hexoside 1 ; **10**= Apigenin pentosyl hexoside 2; **11**= Apigenin pentosyl hexoside 3; **12**= Luteolin-8-C-hexoside 1; **13**= Luteolin-8-C-hexoside 2; **14**= Kaempferol dihexoside; **15**= Quercetin rutinoside ; **16**= Quercetin rhamnoside; **17**= Luteolin-7-O- hexoside; **18**= Isorhamnetin rhamnoside.

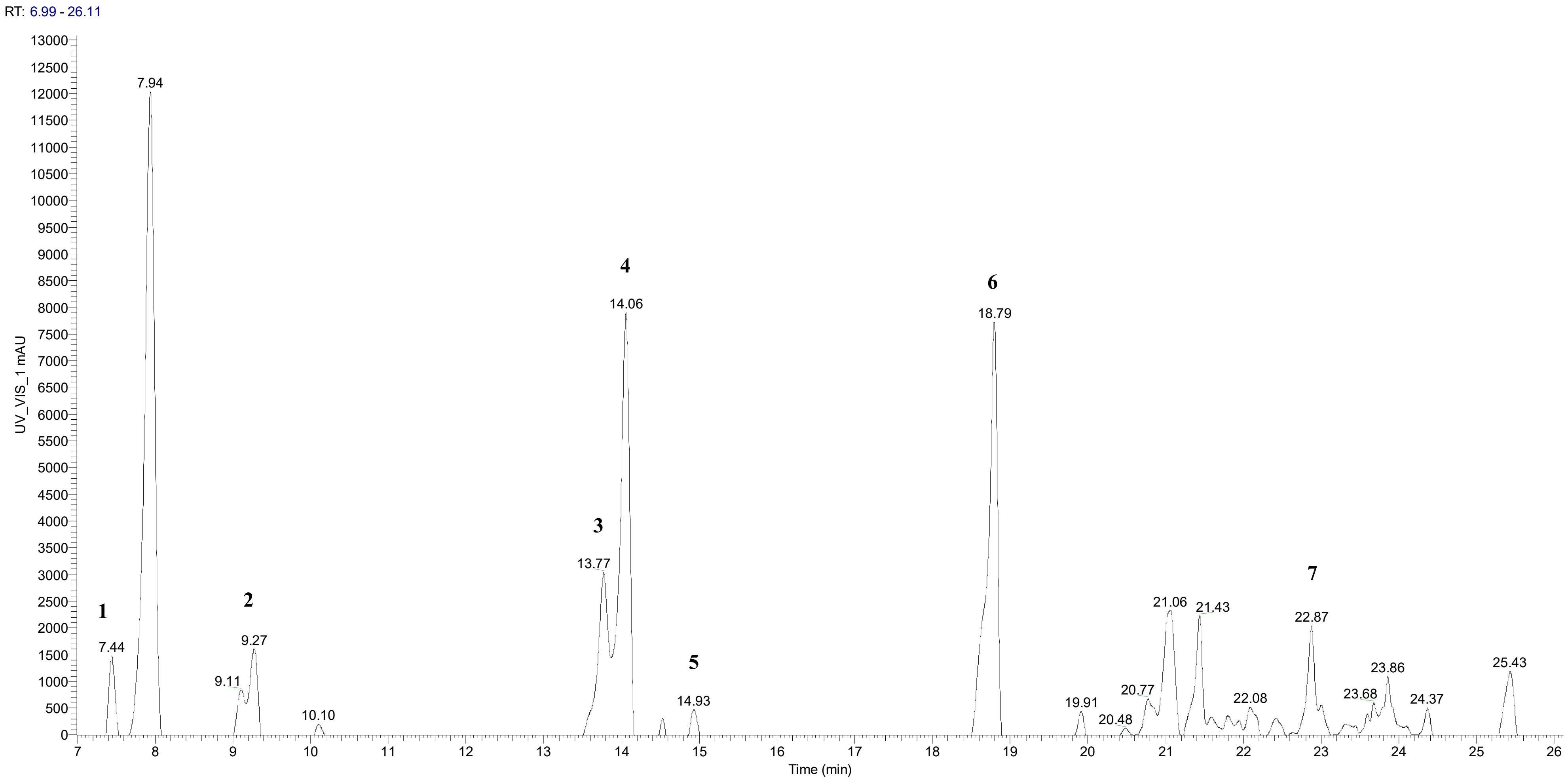

Figure S2. Full scan on a HPLC-MS, and the phenolic compounds identified for placenta. **1**= Coumaroylquinic acid derivative 1; **2**= Tricin; **3**= Caffeic acid hexoside derivative; **4=** Ferulic acid hexoside 1; **5**= Apigenin pentosyl hexoside 1; **6=** Luteolin-8-C-hexoside 1; **7**= Quercetin rhamnoside.

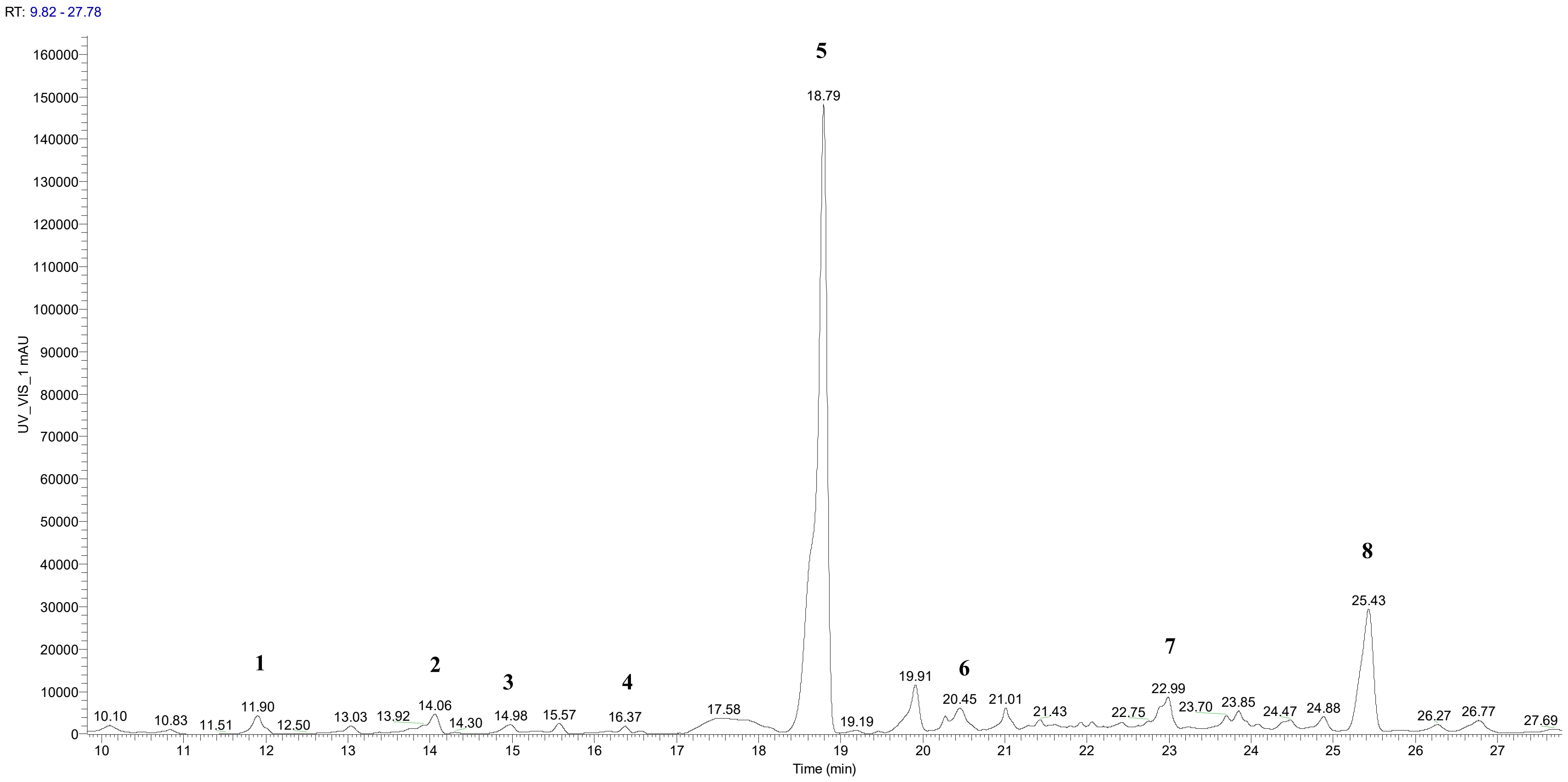

Figure S3. Full scan on a HPLC-MS, and the phenolic compounds identified for seeds. **1**= Caffeic acid hexoside 2; **2**= Ferulic acid hexoside 1; **3**= Ferulic acid hexoside 2; **4=** Caffeic acid hexoside 3; **5**= Luteolin-8-C-hexoside 1; **6=** Luteolin-8-C-hexoside 2; **7**= Kaempferol hexoside 1; **8**= Kaempferol hexoside 2

Table S1. Individual and total phenolics of three fruit parts of the 'Somborka' cultivar under salt stressed (mg 100 g^{-1} DW; mean ± SE).

Cultivar	Fruit part	Phenolics	Treatment						
			Control		20 mM NaCl		40 mM NaCl		Significance
'Somborka'	Pericarp	Coumaroylquinic acid derivative 1	13.91 ± 1.15	c	23.55 ± 4.82	b	35.46 ± 9.37	a	***
		Caffeic acid hexoside 1	4.79 ± 1.10	c	25.37 ± 6.88	b	37.10 ± 2.90	a	***
		Caffeic acid hexoside 2	15.92 ± 4.44	a	16.65 ± 3.68	a	8.93 ± 1.40	a	NS
		p-coumaroylquinic acid	5.67 ± 1.22	c	6.71 ± 1.47	b	7.82 ± 1.08	a	***
		Caffeic acid hexoside derivative	3.60 ± 0.84	c	12.55 ± 3.44	b	45.15 ± 5.28	a	**
		Ferulic acid hexoside 1	1.18 ± 0.24	c	17.42 ± 5.43	b	74.19 ± 4.03	a	***
		Ferulic acid hexoside 2	7.94 ± 0.93	c	7.67 ± 1.11	b	10.18 ± 1.48	a	***
		Chlorogenic acid	5.48 ± 0.98	c	20.40 ± 4.11	b	43.67 ± 1.49	a	***
		**Total analyzed Hydroxycinnamic acids**	**58.49 ± 5.42**	**c**	**130.32 ± 6.28**	**b**	**262.5 ± 3.52**	**a**	***
		Apigenin pentysil hexoside 1	0.04 ± 0.01	c	8.07 ± 3.60	b	14.67 ± 3.56	a	***
		Apigenin pentosyl hexoside 2	11.56 ± 1.09	a	13.35 ± 1.96	a	12.93 ± 4.55	a	NS
		Apigenin pentosyl hexoside 3	17.62 ± 2.18	c	29.29 ± 1.24	b	59.18 ± 4.29	a	***
		Luteolin-8-C-hexoside 1	185.64 ± 4.97	c	281.02 ± 7.28	b	366.11 ± 8.35	a	**
		Luteolin-8-C-hexoside 2	13.99 ± 6.99	c	112.64 ± 13.77	b	151.90 ± 26.37	a	***
		Luteolin-7-*O*- hexoside	699.42 ± 35.10	c	968.03 ± 68.98	b	1190.90 ± 75.83	a	***

	**Total analyzed Flavone**	**916.66 ± 10.63**	**c**	**1412.41 ± 34.63**	**b**	**1795.70 ± 37.65**	**a**	***
	Kaempferol dihexoside	3.60 ± 9.43	c	8.47 ± 0.41	b	9.43 ± 0.85	a	*
	Quercetin rutinoside	7.14 ± 1.63	a	32.75 ± 4.55	a	19.63 ± 1.98	a	NS
	Quercetin rhamnoside	255.50 ± 4.22	c	362.60 ± 5.22	b	304.22 ± 2.48	a	**
	Isoramnetin rhamnoside	23.17 ± 4.12	c	26.81 ± 1.23	b	34.14 ± 3.00	a	***
	**Total analyzed Flavonols**	**289.41 ± 20.63**	**c**	**430.63 ± 32.59**	**b**	**367.42 ± 29.86**	**a**	***
	**Total analyzed phenolics**	**1264.56 ± 43.63**	**c**	**1973.36 ± 99.60**	**b**	**2425.62 ± 123.83**	**a**	***
Placenta	Coumaroylquinic acid derivative 1	65.47 ± 1.31	**a**	29.69 ± 1.56	**c**	53.94 ± 5.92	**b**	***
	Caffeic acid hexoside derivative	133.74 ± 6.32	**a**	25.18 ± 1.17	**c**	71.84 ± 6.35	**b**	***
	Ferulic acid hexoside 1	182.60 ± 14.86	**a**	31.90 ± 5.73	**c**	119.18 ± 9.78	**b**	*
	**Total analyzed Hydroxycinnamic acids**	**381.81 ± 22.62**	**a**	**86.77 ± 8.55**	**c**	**244.96 ± 9.42**	**b**	***
	Apigenin pentysil hexoside 1	54.18 ± 2.50	**a**	36.99 ± 1.05	**a**	42.01 ± 6.90	**a**	NS
	Luteolin-8-C-hexoside 1	317.26 ± 4.94	**a**	106.88 ± 4.72	**c**	219.63 ± 8.31	**b**	**
	Tricin	328.68 ± 16.28	**a**	229.13 ± 7.25	**a**	389.80 ± 4.65	**a**	NS
	**Total analyzed Flavone**	**700.11 ± 65.43**	**a**	**372.99 ± 21.35**	**c**	**651.44 ± 23.55**	**b**	***
	Quercetin rhamnoside	31.46 ± 1.32	**a**	19.77 ± 7.78	**a**	71.47 ± 6.70	**a**	NS
	**Total analyzed Flavonols**	**31.46 ± 1.32**	**a**	**19.77 ± 7.78**	**a**	**71.47 ± 6.70**	**a**	NS
	**Total analyzed phenolics**	**1133.28 ± 78.72**	**a**	**512.06 ± 60.09**	**c**	**1008.99 ± 45.49**	**b**	***
Seeds	Caffeic acid hexoside 2	149.61 ± 5.75	**a**	201.24 ± 2.19	**a**	111.15 ± 1.24	**a**	NS
	Ferulic acid hexoside 1	25.03 ± 5.22	**b**	53.98 ± 7.13	**a**	50.92 ± 6.03	**a**	***
	Ferulic acid hexoside 2	34.77 ± 22.42	**a**	59.59 ± 17.99	**a**	37.64 ± 1.58	**a**	NS

Caffeic acid hexoside 3	598.91 ± 18.43	**b**	1421.31 ± 98.25	**a**	1488.02 ± 35.89	**a**	***
**Total analyzed Hydroxycinnamic acids**	**808.31 ± 43.42**	**b**	**1736.12 ± 53.66**	**a**	**1687.73 ± 26.45**	**a**	***
Luteolin-8-C-hexoside 1	1016.98 ± 49.40	**b**	2410.33 ± 44.89	**a**	1135.74 ± 47.41	**ab**	**
Luteolin-8-C-hexoside 2	102.20 ± 3.34	**c**	148.43 ± 9.61	**b**	758.22 ± 49.82	**a**	***
**Total analyzed Flavone**	**1119.18 ± 56.53**	**c**	**2558.76 ± 63.98**	**a**	**1893.96 ± 47.86**	**b**	**
Kempferol hexoside 1	13.71 ± 3.64	**c**	18.65 ± 6.58	**b**	206.72 ± 25.64	**a**	***
Kempferol hexoside 2	24.39 ± 9.99	**a**	18.25 ± 12.90	**a**	25.19 ± 2.36	**a**	NS
**Total analyzed Flavonols**	**38.09 ± 1.52**	**b**	**36.90 ± 2.31**	**b**	**231.91 ± 10.52**	**a**	***
**Total analyzed phenolics**	**1965.58 ± 58.85**	**c**	**4331.78 ± 77.74**	**a**	**3813.60 ± 73.08**	**b**	***

different letters in the row (a, b, c) indicates statistical differences among treatments Significance codes: *** ≤ 0.001; ** ≤ 0.01; * ≤ 0.05; NS > 0.05

Table S2. Individual and total phenolics of three fruit parts of the 'Novosadka' cultivar under salt stressed (mg 100 g^{-1} DW; mean ± SE).

Cultivar	Fruit part	Phenolics	Treatment						
			Control		20 mM NaCl		40 mM NaCl		Significance
'Novosadka'	Pericarp	Coumaroylquinic acid derivative 1	24.40 ± 9.10	a	20.78 ± 4.36	a	34.83 ± 7.02	a	NS
		Caffeic acid hexoside 1	29.49 ± 1.28	a	17.59 ± 3.28	a	26.91 ± 1.20	a	NS
		Caffeic acid hexoside 2	21.63 ± 4.87	a	11.70 ± 4.56	a	31.87 ± 1.85	a	NS
		p-coumaroylquinic acid	6.13 ± 1.46	a	3.53 ± 0.19	a	8.80 ± 0.84	a	NS
		Caffeic acid hexoside derivative	21.93 ± 5.90	a	19.22 ± 2.29	a	29.11 ± 4.10	a	NS
		Ferulic acid hexoside 1	17.87 ± 5.80	c	19.30 ± 3.84	b	27.05 ± 0.47	a	***
		Ferulic acid hexoside 2	4.59 ± 0.14	a	8.85 ± 3.26	a	6.40 ± 3.16	a	NS
		Chlorogenic acid	21.40 ± 4.74	a	30.09 ± 7.37	a	22.39 ± 2.70	a	NS
		**Total analyzed Hydroxycinnamic acids**	**147.49 ± 2.16**	**a**	**131.06 ± 3.17**	**a**	**187.36± 2.63**	**a**	NS
		Apigenin pentysil hexoside 1	5.97 ± 1.07	a	12.95 ± 3.63	a	6.78 ± 0.53	a	NS
		Apigenin pentosyl hexoside 2	2.77 ± 0.66	a	5.18 ± 1.99	a	0.96 ± 0.20	a	NS
		Apigenin pentosyl hexoside 3	13.43 ± 4.56	a	22.12 ± 2.25	a	20.97 ± 5.23	a	NS
		Luteolin-8-C-hexoside 1	89.15 ± 6.57	a	111.50 ± 21.97	a	106.26 ± 12.92	a	NS
		Luteolin-8-C-hexoside 2	81.98 ± 7.98	a	87.93 ± 18.05	a	67.05 ± 7.77	a	NS
		Luteolin-7-*O*- hexoside	644.07 ± 25.65	a	508.69 ± 11.18	a	568.56 ± 27.01	a	NS
		**Total analyzed Flavone**	**837.36 ± 34.72**	**a**	**748.38 ± 45.24**	**a**	**770.58 ± 44.22**	**a**	NS

	Kaempferol dihexoside	7.75 ± 2.89	a	2.80 ± 0.24	a	2.63 ± 0.94	a	NS
	Quercetin rutinoside	14.81 ± 3.16	a	7.52 ± 0.24	a	7.78 ± 0.45	a	NS
	Quercetin rhamnoside	163.72 ± 6.81	a	129.32 ± 5.98	a	227.23 ± 5.32	a	NS
	Isoramnetin rhamnoside	30.76 ± 4.69	a	14.26 ± 4.57	a	23.87 ± 1.24	a	NS
	**Total analyzed Flavonols**	**217.04 ± 7.47**	**a**	**153.90 ± 9.60**	**a**	**261.51 ± 13.52**	**a**	NS
	**Total analyzed phenolics**	**1201.89 ± 64.99**	**a**	**1033.34 ± 43.57**	**a**	**1219.45 ± 54.74**	**a**	NS
Placenta	Coumaroylquinic acid derivative 1	39.33 ± 14.07	**a**	52.23 ± 16.08	**a**	85.52 ± 11.23	**a**	NS
	Caffeic acid hexoside derivative	87.10 ± 11.61	**a**	196.82 ± 58.70	**a**	267.66 ± 31.88	**a**	NS
	Ferulic acid hexoside 1	155.61 ± 46.88	**a**	259.49 ± 79.09	**a**	387.70 ± 43.99	**a**	NS
	**Total analyzed Hydroxycinnamic acids**	**282.04 ± 21.09**	**c**	**508.54 ± 20.67**	**b**	**740.88 ± 32.63**	**a**	***
	Apigenin pentysil hexoside 1	28.81 ± 4.88	**a**	53.83 ± 16.32	**a**	41.53 ± 8.20	**a**	NS
	Luteolin-8-C-hexoside 1	586.04 ± 245.96	**b**	734.40 ± 119.81	**a**	544.52 ± 28.21	**b**	**
	Tricin	524.95 ± 207.03	**b**	517.74 ± 151.90	**b**	812.36 ± 69.18	**a**	***
	**Total analyzed Flavone**	**1139.79 ± 32.75**	**b**	**1305.97 ± 54.77**	**a**	**1398.40 ± 65.22**	**a**	***
	Quercetin rhamnoside	17.32 ± 4.38	**a**	20.07 ± 7.89	**a**	37.65 ± 30.04	**a**	NS
	**Total analyzed Flavonols**	**17.32 ± 4.38**	**a**	**20.07 ± 7.89**	**a**	**37.65 ± 30.04**	**a**	NS
	**Total analyzed phenolics**	**1493.15 ± 24.63**	**c**	**1834.58 ± 45.74**	**b**	**2176.93 ± 87.36**	**a**	***
Seeds	Caffeic acid hexoside 2	31.10 ± 5.07	**a**	20.29 ± 0.73	**a**	30.61 ± 5.28	**a**	NS
	Ferulic acid hexoside 1	8.19 ± 5.39	**c**	22.31 ± 3.76	**b**	43.45 ± 15.14	**a**	***
	Ferulic acid hexoside 2	8.10 ± 2.29	**a**	7.82 ± 2.51	**a**	8.44 ± 0.84	**a**	NS
	Caffeic acid hexoside 3	28.60 ± 10.63	**a**	21.99 ± 11.04	**a**	12.98 ± 5.55	**a**	NS

**Total analyzed Hydroxycinnamic acids**	**75.99 ± 1.42**	**b**	**72.40 ± 1.46**	**b**	**95.48 ± 1.72**	**a**	***
Luteolin-8-C-hexoside 1	2313.24 ± 852.99	**b**	2370.60 ± 238.65	**b**	2733.34 ± 186.26	**a**	***
Luteolin-8-C-hexoside 2	240.19 ± 17.84	**a**	176.87 ± 26.01	**a**	239.70 ± 59.71	**a**	NS
**Total analyzed Flavone**	**2553.42 ± 437.36**	**b**	**2547.47 ± 532.47**	**b**	**2973.03 ± 456.73**	**a**	**
Kempferol hexoside 1	21.09 ± 10.09	**a**	19.42 ± 3.51	**a**	22.60 ± 5.03	**a**	NS
Kempferol hexoside 2	176.61 ± 13.49	**a**	161.83 ± 0.87	**a**	129.28 ± 20.90	**a**	NS
**Total analyzed Flavonols**	**197.69 ± 2.52**	**a**	**181.25 ± 2.04**	**a**	**151.87 ± 2.01**	**a**	NS
**Total analyzed phenolics**	**2827.11 ± 232.52**	**b**	**2801.13 ± 321.53**	**b**	**3220.40 ± 432.42**	**a**	***

different letters in the row (a, b, c) indicates statistical differences among treatments Significance codes: *** ≤ 0.001; ** ≤ 0.01; * ≤ 0.05; NS > 0.05

Table S3. Individual and total phenolics of three fruit parts of the 'Berenyi F1' cultivar under salt stressed (mg 100 g^{-1} DW; mean ± SE).

Cultivar	Fruit part	Phenolics	Treatment						
			Control		20 mM NaCl		40 mM NaCl		Significance
'Berenyi F1'	Pericarp	Coumaroylquinic acid derivative 1	9.83 ± 2.05	a	10.10 ± 2.77	a	9.16 ± 3.23	a	NS
		Caffeic acid hexoside 1	33.22 ± 5.01	b	46.35 ± 4.34	a	43.35 ± 6.09	a	**
		Caffeic acid hexoside 2	10.77 ± 4.33	a	8.11 ± 4.11	a	19.15 ± 5.20	a	NS
		p-coumaroylquinic acid	6.52 ± 1.33	a	5.82 ± 0.96	a	10.61 ± 2.69	a	NS
		Caffeic acid hexoside derivative	53.16 ± 7.02	a	28.27 ± 13.90	a	54.15 ± 10.31	a	NS
		Ferulic acid hexoside 1	71.96 ± 10.61	a	28.73 ± 11.44	b	82.94 ± 14.48	a	***
		Ferulic acid hexoside 2	5.49 ± 1.09	a	3.94 ± 1.75	a	3.57 ± 0.75	a	NS
		Chlorogenic acid	16.65 ± 4.00	b	58.03 ± 40.07	a	13.14 ± 5.51	b	***
		**Total analyzed Hydroxycinnamic acids**	**207.60 ± 10.77**	**b**	**189.35 ± 9.84**	**b**	**236.07 ± 12.63**	**a**	***
		Apigenin pentysil hexoside 1	8.71 ± 2.79	a	10.01 ± 1.63	a	7.39 ± 1.44	a	NS
		Apigenin pentosyl hexoside 2	3.10 ± 0.20	a	8.53 ± 3.43	a	2.59 ± 0.85	a	NS
		Apigenin pentosyl hexoside 3	18.49 ± 2.15	a	19.45 ± 11.95	a	21.57 ± 4.18	a	NS
		Luteolin-8-C-hexoside 1	230.46 ± 50.78	a	129.77 ± 63.76	b	88.62 ± 12.72	b	**
		Luteolin-8-C-hexoside 2	197.93 ± 2.96	a	155.58 ± 5.60	a	174.01 ± 4.85	a	NS
		Luteolin-7-*O*- hexoside	254.93 ± 2.44	c	353.62 ± 9.38	b	416.46 ± 44.77	a	***
		**Total analyzed Flavone**	**713.61 ± 21.08**	**a**	**676.95 ± 10.63**	**a**	**710.64 ± 9.75**	**a**	NS
		Kaempferol dihexoside	2.22 ± 0.74	a	5.24 ± 2.80	a	0.74 ± 0.51	a	NS

	Quercetin rutinoside	N/D		N/D		N/D		NS
	Quercetin rhamnoside	92.27 ± 7.37	a	121.64 ± 9.29	a	107.29 ± 4.12	a	NS
	Isoramnetin rhamnoside	26.84 ± 1.79	b	35.31 ± 7.33	a	34.87 ± 7.26	a	***
	**Total analyzed Flavonols**	**121.33 ± 1.75**	**a**	**162.19 ± 2.07**	**a**	**142.90± 1.88**	**a**	NS
	**Total analyzed phenolics**	**1042.54 ± 67.73**	**a**	**1028.49 ± 59.34**	**a**	**1089.61 ± 44.64**	**a**	NS
Placenta	Coumaroylquinic acid derivative 1	51.87 ± 16.65	**a**	31.29 ± 5.24	**a**	25.41 ± 7.27	**a**	NS
	Caffeic acid hexoside derivative	181.90 ± 32.42	**a**	220.74 ± 20.36	**a**	180.99 ± 36.84	**a**	NS
	Ferulic acid hexoside 1	250.04 ± 34.79	**a**	283.25 ± 39.73	**a**	265.40 ± 35.34	**a**	NS
	**Total analyzed Hydroxycinnamic acids**	**483.81 ± 23.63**	**a**	**535.28 ± 33.58**	**a**	**471.80 ± 48.00**	**a**	NS
	Apigenin pentysil hexoside 1	33.46 ± 3.08	**a**	35.83 ± 6.50	**a**	27.14 ± 1.42	**a**	NS
	Luteolin-8-C-hexoside 1	648.93 ± 53.20	**a**	806.15 ± 88.19	**a**	659.29 ± 28.70	**a**	NS
	Tricin	386.81 ± 107.47	**a**	271.96 ± 47.16	**a**	404.62 ± 31.77	**a**	NS
	**Total analyzed Flavone**	**1069.20 ± 25.42**	**a**	**1113.93 ± 54.58**	**a**	**1091.04 ± 39.64**	**a**	NS
	Quercetin rhamnoside	27.44 ± 21.84	**a**	28.79 ± 18.24	**a**	9.07 ± 2.02	**a**	NS
	**Total analyzed Flavonols**	**27.44 ± 21.84**	**a**	**28.79 ± 18.24**	**a**	**9.07 ± 2.02**	**a**	NS
	**Total analyzed phenolics**	**1580.45 ± 133.49**	**a**	**1678.00 ± 143.64**	**a**	**1571.91 ± 166.63**	**a**	NS
Seeds	Caffeic acid hexoside 2	23.02 ± 2.08	**a**	21.75 ± 1.18	**a**	22.81 ± 0.91	**a**	NS
	Ferulic acid hexoside 1	10.20 ± 3.63	**a**	9.07 ± 1.46	**a**	25.23 ± 10.41	**a**	NS
	Ferulic acid hexoside 2	6.27 ± 0.08	**a**	6.27 ± 0.35	**a**	6.59 ± 0.64	**a**	NS
	Caffeic acid hexoside 3	6.16 ± 1.82	**a**	7.08 ± 1.26	**a**	6.61 ± 1.81	**a**	NS
	**Total analyzed Hydroxycinnamic acids**	**45.64 ± 1.43**	**a**	**44.17 ± 1.50**	**b**	**61.23 ± 1.67**	**a**	***

Luteolin-8-C-hexoside 1	3687.84 ± 229.04	**a**	4177.32 ± 613.57	**a**	3880.09 ± 365.23	**a**	NS
Luteolin-8-C-hexoside 2	140.93 ± 16.31	**a**	130.35 ± 10.79	**a**	167.68 ± 19.50	**a**	NS
**Total analyzed Flavone**	**3828.76 ± 284.84**	**b**	**4307.66 ± 363.60**	**a**	**4047.76 ± 401.46**	**ab**	**
Kempferol hexoside 1	37.02 ± 1.11	**a**	33.08 ± 0.94	**a**	40.03 ± 4.67	**a**	NS
Kempferol hexoside 2	157.73 ± 2.00	**a**	131.60 ± 9.54	**a**	149.43 ± 4.21	**a**	NS
**Total analyzed Flavonols**	**194.75 ± 2.47**	**a**	**164.67 ± 1.99**	**a**	**189.45 ± 1.68**	**a**	NS
**Total analyzed phenolics**	**4069.15 ± 305.47**	**b**	**4516.51 ± 436.42**	**a**	**4298.46 ± 399.47**	**a**	***

N/D = not determined; different letters in the row (a, b, c) indicates statistical differences among treatments Significance codes: *** ≤ 0.001; ** ≤ 0.01; * ≤ 0.05; NS > 0.05

Table S4. Individual and total phenolics of three fruit parts of the 'Caro F1' cultivar under salt stressed (mg 100 g^{-1} DW; mean ± SE).

Cultivar	Fruit part	Phenolics	Treatment						
			Control		20 mM NaCl		40 mM NaCl		Significance
'Caro F1'	Pericarp	Coumaroylquinic acid derivative 1	13.57 ± 2.35	c	38.56 ± 2.35	a	16.89 ± 9.52	b	**
		Caffeic acid hexoside 1	9.98 ± 0.59	b	24.16 ± 7.78	a	21.84 ± 4.11	a	***
		Caffeic acid hexoside 2	16.23 ± 2.24	a	31.46 ± 5.98	a	34.18 ± 3.85	a	NS
		p-coumaroylquinic acid	2.91 ± 1.22	c	13.86 ± 6.51	b	23.52 ± 6.68	a	***
		Caffeic acid hexoside derivative	19.88 ± 4.91	b	30.07 ± 3.62	a	27.69 ± 8.31	a	***
		Ferulic acid hexoside 1	10.94 ± 2.48	b	31.37 ± 1.64	a	27.84 ± 1.32	a	***
		Ferulic acid hexoside 2	2.73 ± 1.66	b	4.10 ± 0.99	a	5.59 ± 0.30	a	***
		Chlorogenic acid	11.76 ± 2.78	c	16.96 ± 6.70	b	36.79 ± 4.42	a	***
		**Total analyzed Hydroxycinnamic acids**	**88.00 ± 2.68**	**b**	**190.54 ± 10.74**	**a**	**194.34 ± 10.85**	**a**	***
		Apigenin pentysil hexoside 1	16.80 ± 7.92	a	14.50 ± 9.55	a	7.09 ± 1.75	a	NS
		Apigenin pentosyl hexoside 2	10.98 ± 6.99	a	7.94 ± 1.83	a	17.52 ± 4.84	a	NS
		Apigenin pentosyl hexoside 3	16.10 ± 4.86	a	18.73 ± 1.68	a	37.89 ± 14.77	a	NS
		Luteolin-8-C-hexoside 1	176.30 ± 16.53	c	284.46 ± 117.92	b	379.29 ± 26.33	a	**
		Luteolin-8-C-hexoside 2	137.21 ± 26.78	a	173.00 ± 55.01	a	133.23 ± 26.51	a	NS
		Luteolin-7-*O*- hexoside	396.20 ± 28.31	c	751.76 ± 50.17	b	924.38 ± 17.86	a	***
		**Total analyzed Flavone**	**753.60 ± 5.53**	**c**	**1250.38 ± 97.63**	**b**	**1499.40 ± 101.57**	**a**	***
		Kaempferol dihexoside	2.50 ± 3.60	c	5.01 ± 8.47	b	8.98 ± 9.43	a	**

	Quercetin rutinoside	43.33 ± 7.14	c	60.74 ± 32.75	b	128.39 ± 19.63	a	***
	Quercetin rhamnoside	123.04 ± 25.50	c	321.61 ± 32.60	b	432.93 ± 34.22	a	**
	Isoramnetin rhamnoside	17.74 ± 5.52	c	29.18 ± 7.63	b	39.35 ± 1.50	a	***
	**Total analyzed Flavonols**	**186.61 ± 1.24**	**c**	**416.54 ± 2.46**	**b**	**609.65 ± 5.99**	**a**	***
	**Total analyzed phenolics**	**1028.21 ± 78.89**	**c**	**1857.46 ± 99.47**	**b**	**2303.39 ± 127.54**	**a**	***
Placenta	Coumaroylquinic acid derivative 1	16.59 ± 1.03	**c**	158.81 ± 8.37	**a**	66.05 ± 8.46	**b**	***
	Caffeic acid hexoside derivative	64.21 ± 11.89	**a**	128.64 ± 25.28	**a**	82.99 ± 5.57	**a**	NS
	Ferulic acid hexoside 1	79.10 ± 32.20	**a**	145.10 ± 46.39	**a**	74.58 ± 8.88	**a**	NS
	**Total analyzed Hydroxycinnamic acids**	**159.90 ± 11.53**	**b**	**432.55 ± 43.60**	**a**	**223.62 ± 32.45**	**b**	***
	Apigenin pentysil hexoside 1	34.46 ± 15.25	**a**	52.95 ± 18.00	**a**	28.20 ± 5.41	**a**	NS
	Luteolin-8-C-hexoside 1	804.98 ± 88.34	**a**	436.74 ± 40.89	**b**	519.22 ± 120.73	**b**	***
	Tricin	366.43 ± 39.89	**a**	325.15 ± 17.15	**a**	217.76 ± 45.34	**a**	NS
	**Total analyzed Flavone**	**1205.87 ± 77.34**	**a**	**814.83 ± 54.42**	**b**	**765.19 ± 43.42**	**b**	***
	Quercetin rhamnoside	75.23 ± 37.70	**a**	23.77 ± 14.83	**b**	30.21 ± 15.71	**b**	***
	**Total analyzed Flavonols**	**75.22 ± 6.52**	**a**	**23.76 ± 1.08**	**b**	**30.21 ± 1.99**	**b**	***
	**Total analyzed phenolics**	**1440.99 ± 77.74**	**a**	**1271.14 ± 84.25**	**b**	**1019.02 ± 25.55**	**b**	***
Seeds	Caffeic acid hexoside 2	18.10 ± 1.74	**a**	19.94 ± 1.69	**a**	15.32 ± 2.38	**a**	NS
	Ferulic acid hexoside 1	7.44 ± 1.93	**b**	12.08 ± 4.06	**a**	15.64 ± 2.22	**a**	**
	Ferulic acid hexoside 2	4.50 ± 0.82	**a**	6.20 ± 0.31	**a**	6.35 ± 0.59	**a**	NS
	Caffeic acid hexoside 3	6.22 ± 3.32	**a**	6.69 ± 0.36	**a**	9.92 ± 6.25	**a**	NS
	**Total analyzed Hydroxycinnamic acids**	**36.25 ± 1.75**	**b**	**44.90 ± 2.77**	**a**	**47.23 ± 2.52**	**a**	***

Luteolin-8-C-hexoside 1	2269.96 ± 60.15	**a**	1486.43 ± 214.45	**b**	1090.42 ± 92.76	**c**	***
Luteolin-8-C-hexoside 2	155.85 ± 17.91	**a**	267.99 ± 50.61	**a**	241.51 ± 28.13	**a**	NS
**Total analyzed Flavone**	**2425.80 ± 43.64**	a	**1754.42 ± 50.65**	b	**1331.92 ± 54.35**	c	***
Kempferol hexoside 1	33.51 ± 7.00	**a**	15.79 ± 5.05	**b**	14.63 ± 2.89	**b**	***
Kempferol hexoside 2	89.25 ± 11.46	**a**	84.23 ± 19.70	**a**	64.58 ± 16.06	**a**	NS
**Total analyzed Flavonols**	**122.75 ± 5.67**	a	**100.02 ± 5.09**	b	**79.20 ± 2.53**	b	***
**Total analyzed phenolics**	**2584.82 ± 452.36**	a	**1899.34 ± 325.50**	b	**1458.36 ± 408.74**	c	***

different letters in the row (a, b, c) indicates statistical differences among treatments Significance codes: *** ≤ 0.001; ** ≤ 0.01; * ≤ 0.05; NS > 0.05

Table S5. Individual and total capsaicnoid contents of three fruit parts of the four cultivars under salt stressed (mg 100 g^{-1} DW; mean ± SE).

		Treatment / capsaicinoid	Treatment Control		20 mM NaCl		40 mM NaCl		Signifcance
**'Somborka'**	Pericarp	Capsaicin	0.02 ± 0.01	c	1.86 ± 0.06	b	2.67 ± 0.24	a	***
		Dihydrocapsaicin	0.01 ± 0.00	c	0.28 ± 0.05	a	0.31 ± 0.08	a	***
		Nordihydrocapsaicin	N/D		N/D		N/D		NS
		Homocapsaicin	N/D		N/D		N/D		NS
		Homodihydrocapsaicin	N/D		N/D		N/D		NS
		**Total capsaicinoids**	**0.03 ± 0.01**	**c**	**2.15 ± 0.13**	**b**	**2.99 ± 0.53**	**a**	***
	Placenta	Capsaicin	0.19 ± 0.04	c	14.39 ± 1.95	b	202.77 ± 2.80	a	***
		Dihydrocapsaicin	0.05 ± 0.02	c	2.51 ± 0.40	b	25.85 ± 5.71	a	***
		Nordihydrocapsaicin	0.00 ± 0.00	c	0.12 ± 0.03	b	0.55 ± 0.55	a	**
		Homocapsaicin	0.02 ± 0.01	c	0.47 ± 0.14	b	3.42 ± 3.42	a	***
		Homodihydrocapsaicin	0.00 ± 0.00	c	0.14 ± 0.02	b	0.99 ± 0.98	a	***
		**Total capsaicinoids**	**0.26 ± 0.07**	**c**	**17.64 ± 2.54**	**b**	**233.58 ± 3.45**	**a**	***
	Seeds	Capsaicin	0.54 ± 0.20	c	3.82 ± 0.30	a	2.04 ± 1.08	b	***
		Dihydrocapsaicin	0.06 ± 0.01	c	0.62 ± 0.04	a	0.33 ± 0.16	b	***
		Nordihydrocapsaicin	0.00 ± 0.00	b	0.03 ± 0.00	a	0.01 ± 0.01	a	***
		Homocapsaicin	0.02 ± 0.01	b	0.05 ± 0.03	a	0.04 ± 0.03	a	***
		Homodihydrocapsaicin	N/D		N/D		N/D		NS

		**Total capsaicinoids**	**0.62 ± 0.22**	**c**	**4.52 ± 0.22**	**a**	**2.44 ± 1.30**	**b**	***
'Novosadka'	Pericarp	Capsaicin	4.32 ± 1.14	c	73.35 ± 1.36	b	195.95 ± 7.75	a	***
		Dihydrocapsaicin	0.61 ± 0.15	c	11.17 ± 3.60	b	38.55 ± 2.33	a	***
		Nordihydrocapsaicin	0.04 ± 0.01	c	0.48 ± 0.17	b	1.85 ± 0.06	a	***
		Homocapsaicin	0.07 ± 0.03	c	3.26 ± 1.21	b	11.74 ± 0.43	a	***
		Homodihydrocapsaicin	0.02 ± 0.00	c	0.87 ± 0.42	b	4.06 ± 0.12	a	***
		**Total capsaicinoids**	**5.06 ± 1.34**	**c**	**89.12 ± 6.77**	**b**	**252.15 ± 10.70**	**a**	***
	Placenta	Capsaicin	27.11 ± 2.60	c	184.87 ± 6.90	b	526.04 ± 3.88	a	***
		Dihydrocapsaicin	5.44 ± 0.60	c	42.54 ± 12.34	b	118.07 ± 9.98	a	***
		Nordihydrocapsaicin	0.41 ± 0.09	c	1.92 ± 0.35	b	5.61 ± 0.37	a	***
		Homocapsaicin	0.81 ± 0.04	c	6.63 ± 1.93	b	28.58 ± 1.88	a	***
		Homodihydrocapsaicin	0.38 ± 0.07	c	3.17 ± 0.45	b	9.95 ± 0.48	a	***
		**Total capsaicinoids**	**34.15 ± 3.38**	**c**	**239.12 ± 6.97**	**b**	**688.25 ± 4.59**	**a**	***
	Seeds	Capsaicin	13.57 ± 2.52	c	116.41 ± 2.39	b	388.97 ± 4.24	a	***
		Dihydrocapsaicin	3.06 ± 0.73	c	29.96 ± 6.90	b	120.05 ± 10.24	a	***
		Nordihydrocapsaicin	0.20 ± 0.05	c	1.47 ± 0.39	b	7.11 ± 0.57	a	***
		Homocapsaicin	0.64 ± 0.14	c	4.95 ± 1.65	b	32.58 ± 3.77	a	**
		Homodihydrocapsaicin	0.19 ± 0.06	c	1.93 ± 0.60	b	13.35 ± 1.69	a	***
		**Total capsaicinoids**	**17.67 ± 3.50**	**c**	**154.71 ± 30.93**	**b**	**562.05 ± 50.50**	**a**	***
'Berenyi F1'	Pericarp	Capsaicin	8.47 ± 2.28	b	20.95 ± 14.49	a	18.09 ± 5.42	a	***
		Dihydrocapsaicin	1.19 ± 0.34	b	2.86 ± 2.13	a	2.93 ± 1.01	a	***
		Nordihydrocapsaicin	0.06 ± 0.03	b	0.18 ± 0.16	a	0.25 ± 0.13	a	**

		Homocapsaicin	0.48 ± 0.15	c	0.76 ± 0.44	b	1.27 ± 0.54	a	***
		Homodihydrocapsaicin	0.26 ± 0.03	a	0.29 ± 0.20	a	0.55 ± 0.26	a	NS
		**Total capsaicinoids**	**10.46 ± 2.83**	**b**	**25.05 ± 17.43**	**a**	**23.08 ± 7.36**	**a**	***
	Placenta	Capsaicin	201.54 ± 7.41	a	114.84 ± 6.68	a	173.41 ± 3.84	a	NS
		Dihydrocapsaicin	38.96 ± 13.78	a	18.67 ± 8.07	a	30.39 ± 3.13	a	NS
		Nordihydrocapsaicin	4.08 ± 2.22	a	1.91 ± 0.86	a	1.83 ± 0.37	a	NS
		Homocapsaicin	15.71 ± 4.28	a	7.87 ± 3.12	a	12.95 ± 1.78	a	NS
		Homodihydrocapsaicin	10.79 ± 5.33	a	5.30 ± 2.30	a	4.61 ± 0.64	a	NS
		**Total capsaicinoids**	**271.09 ± 9.02**	**a**	**148.59 ± 6.02**	**a**	**223.19 ± 29.77**	**a**	NS
	Seeds	Capsaicin	27.89 ± 4.57	b	29.30 ± 3.14	ab	42.92 ± 9.73	a	***
		Dihydrocapsaicin	4.88 ± 1.13	b	4.06 ± 0.38	b	11.13 ± 3.97	a	***
		Nordihydrocapsaicin	0.25 ± 0.08	a	0.24 ± 0.08	a	1.34 ± 0.84	a	NS
		Homocapsaicin	1.55 ± 0.19	a	1.27 ± 0.19	a	2.68 ± 0.49	a	NS
		Homodihydrocapsaicin	0.56 ± 0.07	a	0.45 ± 0.08	a	1.94 ± 1.13	a	NS
		**Total capsaicinoids**	**35.14 ± 6.05**	**b**	**35.32 ± 3.87**	**b**	**60.00 ± 16.16**	**a**	***
**'Caro F1'**	Pericarp	Capsaicin	26.23 ± 1.04	c	75.91 ± 7.86	b	119.14 ± 9.46	a	**
		Dihydrocapsaicin	7.09 ± 2.83	c	11.60 ± 7.91	b	28.56 ± 6.79	a	***
		Nordihydrocapsaicin	0.25 ± 0.14	c	0.67 ± 0.38	b	2.57 ± 0.51	a	*
		Homocapsaicin	0.83 ± 0.42	c	2.73 ± 2.03	b	5.07 ± 0.79	a	***
		Homodihydrocapsaicin	0.56 ± 0.28	c	1.03 ± 0.72	b	2.93 ± 0.54	a	***
		**Total capsaicinoids**	**34.96 ± 2.72**	**c**	**91.94 ± 8.90**	**b**	**158.27 ± 8.09**	**a**	**

Placenta	Capsaicin	632.97 ± 12.88	b	1145.97 ± 40.29	a	1223.90 ± 33.16	a	***
	Dihydrocapsaicin	109.24 ± 36.03	c	247.79 ± 86.15	b	344.32 ± 144.34	a	***
	Nordihydrocapsaicin	8.66 ± 2.19	c	28.87 ± 7.17	b	41.30 ± 18.44	a	***
	Homocapsaicin	26.16 ± 12.97	b	73.13 ± 32.37	a	73.89 ± 19.90	a	***
	Homodihydrocapsaicin	13.11 ± 4.47	c	49.03 ± 4.72	b	62.73 ± 9.52	a	***
	**Total capsaicinoids**	**790.15 ± 34.54**	**c**	**1544.80 ± 50.69**	**b**	**1746.14 ± 55.36**	**a**	***
Seeds	Capsaicin	94.27 ± 10.57	c	163.28 ± 6.34	b	199.71 ± 5.94	a	***
	Dihydrocapsaicin	14.27 ± 1.58	c	28.84 ± 8.03	b	56.21 ± 4.05	a	**
	Nordihydrocapsaicin	1.18 ± 0.14	c	3.05 ± 0.63	b	5.92 ± 2.66	a	***
	Homocapsaicin	3.55 ± 0.46	b	8.39 ± 3.86	a	8.67 ± 1.51	a	***
	Homodihydrocapsaicin	1.64 ± 0.04	c	3.69 ± 0.97	b	5.54 ± 2.34	a	***
	**Total capsaicinoids**	**114.90 ± 12.79**	**c**	**207.24 ± 7.83**	**b**	**276.05 ± 7.51**	**a**	***

N/D = not determined; different letters in the row (a, b, c) indicates statistical differences among treatments Significance codes: *** ≤ 0.001; ** ≤ 0.01; * ≤ 0.05; NS > 0.05
